# Real-time kinetics and high-resolution melt curves in single-molecule digital LAMP to differentiate and study specific and non-specific amplification

**DOI:** 10.1093/nar/gkaa099

**Published:** 2020-02-27

**Authors:** Justin C Rolando, Erik Jue, Jacob T Barlow, Rustem F Ismagilov

**Affiliations:** 1 Division of Chemistry and Chemical Engineering, California Institute of Technology 1200 E. California Boulevard, Pasadena, CA 91125, USA; 2 Division of Biology and Biological Engineering, California Institute of Technology 1200 E. California Boulevard, Pasadena, CA 91125, USA

## Abstract

Isothermal amplification assays, such as loop-mediated isothermal amplification (LAMP), show great utility for the development of rapid diagnostics for infectious diseases because they have high sensitivity, pathogen-specificity and potential for implementation at the point of care. However, elimination of non-specific amplification remains a key challenge for the optimization of LAMP assays. Here, using chlamydia DNA as a clinically relevant target and high-throughput sequencing as an analytical tool, we investigate a potential mechanism of non-specific amplification. We then develop a real-time digital LAMP (dLAMP) with high-resolution melting temperature (HRM) analysis and use this single-molecule approach to analyze approximately 1.2 million amplification events. We show that single-molecule HRM provides insight into specific and non-specific amplification in LAMP that are difficult to deduce from bulk measurements. We use real-time dLAMP with HRM to evaluate differences between polymerase enzymes, the impact of assay parameters (e.g. time, rate or florescence intensity), and the effect background human DNA. By differentiating true and false positives, HRM enables determination of the optimal assay and analysis parameters that leads to the lowest limit of detection (LOD) in a digital isothermal amplification assay.

## INTRODUCTION

Isothermal methods, such as loop-mediated isothermal amplification (LAMP), are attractive for nucleic acid amplification tests (NAATs) in point-of-care and limited-resource settings (1,2). LAMP in particular shows promise as a NAAT with fewer hardware requirements compared with polymerase chain reaction (PCR) (3). Despite advancements, the ability to optimize LAMP NAATs for a specific target sequence and primer set (specific to a target organism) remains constrained by a limited understanding of how amplification is affected by myriad factors, including polymerase choice, primer design, temperature, time and ion concentrations. In particular, addressing non-specific amplification remains a core problem as it constrains an assay's limit of detection (LOD). In reactions containing template target molecules, both specific and non-specific amplification reactions may occur. Unlike PCR, LAMP lacks a temperature-gating mechanism, so non-specific reactions consume reagents and compete with specific amplification impacting its kinetics. The presence of non-specific amplicons therefore adversely impacts both the assay's analytical sensitivity (the fewest template molecules that can be detected) and its analytical specificity (ability to detect the target template in the presence of competing reactions). Classifying reactions as either specific or non-specific amplification would therefore be invaluable both during assay optimization and assay deployment in clinical diagnostics.

Substantial research is focused on using isothermal amplification chemistries for diagnosis of infectious disease. For example, chlamydia (caused by the pathogen *Chlamydia trachomatis*, CT) is the most common sexually transmitted infection worldwide, with more than 110 million cases reported annually (4). Diagnosis of CT infections is challenged by a lack of standard symptoms (many infections are asymptomatic) (5) and the presence of mixed flora (particularly in the female reproductive tract) (6). Thus, rapid NAATs with high sensitivity and specificity are critically needed, especially NAATs that can deal with the high levels of host or background DNA likely to be present in clinical samples such as urine samples and swabs (7,8).

Optimizing LAMP for CT and other infectious pathogens requires addressing and reducing non-specific amplification or a method for separating non-specific reactions from specific amplification. Reactions run in bulk (i.e. in a tube) in the absence of template can be informative to provide information on performance of non-specific amplification. Another method to identify non-specific amplification includes mathematical modeling in conjunction with electrophoresis to distinguish between non-specific and specific banding patterns (9). However, in the presence of template, although specific and non-specific reactions occur simultaneously, they cannot be monitored simultaneously. Thus, bulk reactions have three important limitations with regard to assay optimization: (i) differences in the kinetics of specific and non-specific reactions cannot be separated, (ii) rare but significant events, such as early but infrequent non-specific amplification, cannot be easily characterized; and (iii) testing the full design space requires many hundreds of replicates to obtain statistically significant data. To improve an assay's analytical specificity and sensitivity, one strategy is to eliminate the detection of non-specific amplification. In bulk LAMP experiments, non-specific amplification can be excluded from detection by using probes, beacons, FRET or reporter-quencher schemes that show only specific amplification of the target (10–19). Although these methods improve the assay, they do not capture non-specific reactions and thus cannot give insights into the origin of non-specific amplification or the conditions that led to non-specific amplicons. Moreover, probes and beacons do not eliminate non-specific amplification; non-specific amplification still competes for reagents and can limit the extent of the signal generated by specific amplification events (20). Hence, it is highly desirable to distinguish specific from non-specific amplification.

In this study, we combined sequencing and digital single-molecule LAMP (dLAMP) with high-resolution melting temperature (HRM) to probe the fundamental mechanics of amplification reactions. We used dLAMP to extract real-time kinetic information to identify the digital threshold data-processing parameters that minimize non-specific amplification events and elucidate how an interfering molecule impacts amplification. Digital single-molecule methods separating individual amplification events into discrete compartments, eliminating interference among individual amplification events (21,22). Furthermore, digital experiments consist of thousands of reactions that run in parallel and thus provide valuable statistical information (21–23). We used real-time imaging to monitor the kinetics of 20,000 dLAMP reactions per experiment and observe ∼1.2 × 10^6^ reactions in total. We hypothesized that high-resolution melting analysis (HRM) could be a tool for separating specific from non-specific amplification events and for identifying the optimal digital threshold data-processing parameters to distinguish specific and non-specific amplification events (even when an assay is deployed without HRM). To test this hypothesis, we used a dLAMP assay with CT DNA as the target (combined with sequencing to identify the products of bulk reactions) to analyze both specific and non-specific amplification under conditions that include clinically relevant concentrations of background human DNA.

## MATERIALS AND METHODS

### LAMP reagents

IsoAmp I (#B0537S), IsoAmp II (#B0374S), MgSO_4_ (#B1003S), deoxynucleotide solution (#N0447S), Bovine Serum Albumen (BSA, #B9000S0), *Bst* 2.0 (8,000 U/ml, #M0537S) and *Bst* 3.0 (8000 U/ml, #M0374S) were purchased from New England Biolabs (Ipswich, MA, USA). Ambion RNase Cocktail (#AM2286), Ambion nuclease-free water (#AM9932), Invitrogen SYTO 9 (S34854) and Invitrogen ROX Reference Dye (#12223012) were purchased from Thermo Fisher Scientific (Waltham, MA, USA). We found it important to use SYTO 9 dilutions within one week of preparation.

Primers sequences were targeted against the *Chlamydia trachomatis* 23S ribosomal gene using Primer Explorer V5 (Eiken Chemical, Tokyo, Japan) and checked in SnapGene (GSL Biotech, Chicago, IL, USA) to ensure the sequences were in a mutation free region from the available Genebank sequences of CT. Primers were purchased from Integrated DNA Technologies (San Diego, CA, USA) and suspended in nuclease-free water. For all experiments, the final concentrations of primers were 1.6 μM FIP/BIP, 0.2 μM FOP/BOP and 0.4 μM LoopF/LoopB. Primer sequences are listed in Supplementary Materials and Methods.

LAMP experiments using *Bst* 2.0 were amplified at 68°C in nuclease-free water, with final concentrations of: 1× IsoAmp I Buffer, 7mM total MgSO_4_ (5 mM additional), 1.4 mM each dNTP, 1.25 uM ROX Reference Dye, 1 mg/ml BSA, 320 U/ml *Bst* 2.0, 1× Ambion RNase Cocktail and 2 µM SYTO 9.

LAMP experiments using *Bst* 3.0 were amplified at 69°C in nuclease-free water, with final concentrations of: 1× IsoAmp II Buffer, 8 mM total MgSO_4_ (6 mM additional), 1.4 mM each dNTP, 1.25 μM ROX Reference Dye, 1 mg/ml BSA, 320 U/ml *Bst* 3.0, 1× Ambion RNase Cocktail and 2 µM SYTO 9.

For both enzymes, after 90 min of amplification, reactions were ramped to 95°C at maximum output and held for 30 s to inactivate the enzymes. Chips were cooling to 55°C and the melt performed at a ramp rate of 1°C per image from 55–90°C, and a ramp rate of 0.5°C per image from 90–95°C.

### Extraction of spiked *Chlamydia trachomatis* (CT) from a relevant clinical matrix

A frozen stock of live CT (D-UW3, Z054, Zeptometrix, Buffalo, NY, USA) was re-suspended in pre-warmed (37°C) SPG buffer (219 mM sucrose, 3.7 mM KH2PO4, 8.5 mM NA2HPO4, and 4.9 mM L-glutamate) buffer to 1 × 10^8^ IFU/ml. It was then diluted 10-fold into a freshly donated urine sample to 1 × 10^7^ IFU/ml. Urine from a healthy human donor (>18 years of age) was acquired and used in accordance with approved Caltech Institutional Review Board (IRB) protocol 15-0566. Written informed consent was obtained from all participants, donations were never tied to personal identifiers and all research was performed in accordance with relevant institutional biosafety regulations. A 250 μl aliquot from this CT-spiked urine sample was then extracted following the ZR Viral DNA/RNA Kit protocol (#D7020, Zymo Research, Irvine, CA, USA). Briefly, 250 μl of CT-spiked urine was mixed with 250 μl DNA/RNA shield and 1000 μl DNA/RNA Viral Buffer. A total of 1500 μl (750 μl × 2) was added to the column and centrifuged at 16 000 × *g* for 1 min. Then, 500 μl Viral Wash buffer was added to the column and centrifuged at 16 000 × *g* for 2 min. Then, 60 μl DNAse/RNAse-free water was added directly to the column and centrifuged at 16 000 × *g* for 30 s. The eluent was treated by adding 2.5 μl Ambion RNAse Cocktail (#AM2286, ThermoFisher) to 47.5 μl template. Stocks were prepared in 0.5 × TE buffer and dilutions quantified using the QX200 droplet digital PCR system (Bio-Rad Laboratories, Hercules, CA, USA), outer primers at 500 nM each and 1× EvaGreen Supermix (Bio-Rad).

### Fabrication of thermoelectric unit and mount

A Thermoelectric Module (VT-127-1.4-1.5-72), Thermister (MP-3022), Controller (TC-720) and 12V Power Supply (PS-12-8.4; TE Tech, Traverse City, MI, USA) were wired according to the manufacturer's instructions.

While the Peltier can be used out of the box, we manufactured a heat plate and sink to improve the efficiency in the cooling mode. Instructions for fabrication can be found in the Supplementary Materials and Methods, ‘Fabrication of thermoelectric unit mount.’ The ability of the embedded thermocouple to accurately assess temperature of the aluminum block was verified with an independent K-type mini-thermcouple read through a General IRT659K [IR] Thermometer.

### Shearing of genomic DNA

Human genomic DNA from buffy coat leukocytes (Roche (via Sigma Aldrich), #11691112001) was fragmented using a Covaris Focused Ultrasonicator M220 (Woburn, MA, USA) equipped with 130 μL microTUBE AFA Fiber Snap-Cap at 50W peak power, 5% duty factor, 200 cycles per burst, for 80 s. Fragment concentration was determined using a Qbit 3 Fluorimiter (ThermoFisher, #Q33216) with dsDNA HS assay kit (ThermoFisher, #Q32851) and mean fragment size determined as 365 bp using an Agilent 4200 TapeStation (#G2991AA, Agilent, Santa Clara, CA, USA) and High Sensitivity D5000 ScreenTape (#5067-5592) with ladder (#5190-7747), and D100 ScreenTape (#5067-5584) with High Sensitivity D1000 Reagents (#5067-5585). Dilutions were prepared with a final concentration of 0.5× TE buffer.

### Microfluidic chips

Microfluidic chips for dLAMP (#A26316; Applied Biosystems, Foster City, CA, USA) were loaded as we have described previously (23) at a concentration where ∼40% of partitions would fluoresce (corresponding to the Poisson maximum single template per partition loading of 660 cp/μl). We estimated the volume of each partition to be 750 pl. To achieve this concentration of template molecules, we diluted template stocks from storage in 0.5× TE to ∼0.03× TE for all experiments. Genomic DNA (gDNA) stocks, also stored in 0.5× TE, were diluted to a final concentration of 0.077×. Thus, the total final concentration of TE for all experiments of was ∼0.1081× TE buffer.

### Microscopy data collection

Data were collected in 30-s intervals using a DMI-6000B microscope (Leica, Buffalo Grove, IL, USA) equipped with a 1.25 × 0.04NA HCX PL FLUOTAR Objective and 0.55× coupler (Leica C-mount 11541544). The response from SYTO 9 was recorded using a 1.5-s exposure through an L5 (GFP) Nomarski prism, while the ROX Reference Dye was collected using a 1-s exposure through a Texas Red prism. Images were collected using a Hamamatsu ORCA-ER CCD camera (Hamamatsu Photonics K.K., Hamamatsu City, Japan) at 100 gain. Temperature was recoded using the built-in features of the TC-720 Controller in ∼1 s intervals and correlated to the images via image metadata.

In these experiments, we chose to use a microscope, instead of the custom real-time amplification instrument we used previously (23,24), because the microscope has superior optical properties (greater pixels per partition and lower exposure time requirements) to access higher temporal resolution and enhanced kinetic measurements.

### MATLAB script processing

The MATLAB script processes a .txt file with temperature-time data generated from the TE Tech Controller and a TIF stack containing 2-channel images of the LAMP and melt curve from the LEICA microscope. Partitions are identified using a custom iterative thresholding algorithm and labels are propagated throughout the TIF stack using a custom labeling algorithm. Average well intensity is tracked over time to generate LAMP curves and plotted against temperature to generate the melt curves. Complete details of the script are in the Supplementary Materials and Methods, ‘MATLAB script.’

**Bulk LAMP reactions** were conducted in 10 μl volumes within a well plate on a CFX96 Real-time Thermocycler (Bio-Rad) at buffer conditions and temperatures matching the dLAMP reactions.

**Enzymatic digestions** of bulk LAMP products were conducted using CAC8I (#R0579S), Hpy166II (#R0616S), ACCI (#R0161S), AciI (#SR0551S), MseI (#R0525S) and HpyCH4III (#R0618S) purchased from New England Biolabs and were conducted in 50 μl reaction volumes containing 1 μl enzyme, 1 μg DNA, in 1 × Cut Smart Buffer and incubated for 1 h at 37°C. Samples were inactivated for 1 h at 80°C and diluted to 1 ng/μl (∼1:300) to run on an Agilent 4200 TapeStation using High Sensitivity D5000 ScreenTape (#5067-5592) with ladder (#5190-7747), and D100 ScreenTape (#5067-5584) with High Sensitivity D1000 Reagents (#5067-5585).

### Library preparation and sequencing

The 300–500 ng of amplified DNA products were fragmented to the average size of 200 bp with Qsonica Q800R sonicator (power: 20%; pulse: 15 s on/15 s off; sonication time: 12 min) and libraries were constructed using NEBNext Ultra™ II DNA Library Prep Kit (NEB, #E7645) following manufacturer's instructions. Briefly, fragmented DNA was end-repaired, dA tailed and ligated to NEBNext hairpin adaptors (NEB, #E7335). After ligation, adapters were converted to the ‘Y’ shape by treating with USER enzyme and DNA fragments were size selected using Agencourt AMPure XP beads (Beckman Coulter, #A63880) to generate fragment sizes between 250 and 350 bp. Adaptor-ligated DNA was PCR amplified with five cycles followed by AMPure XP bead clean up. Libraries were quantified with Qubit dsDNA HS Kit (ThermoFisher Scientific, #Q32854) and the size distribution was confirmed with High Sensitivity DNA Kit for Bioanalyzer (Agilent Technologies, #5067). Libraries were sequenced on Illumina HiSeq2500 in single-read mode with the read length of 50 nt to the sequencing depth of 10 million reads per sample, following manufacturer's instructions. Base calls were performed with RTA 1.18.64 followed by conversion to FASTQ with bcl2fastq 1.8.4.

### Sequencing analysis

Raw FASTQ files were first analyzed with FastQC v0.11.8. Overrepresented sequences were compared with input primer sequences to find reads consisting of potential products from the LAMP reactions. To verify that all adjoining products were accounted for the FASTQ files were aligned to the predicted products using Bowtie2 v2.3.4.3 with global very-sensitive settings. Unaligned reads were checked for any remaining possible amplification products. All regions consisting of sequences from multiple primers were tallied by counting the reads with a substring of *n* = 11 from the end of each primer. One adjoining region between primers contained a random insertion of nucleotides and was analyzed by first extracting all reads containing the primer before and after the random nucleotides. The length and sequence distribution of random inserts was then analyzed from the extracted reads.

## RESULTS AND DISCUSSION

### Bulk LAMP studies reveal non-specific products with high melting temperature (*T*_m_)

**Figure 1.**
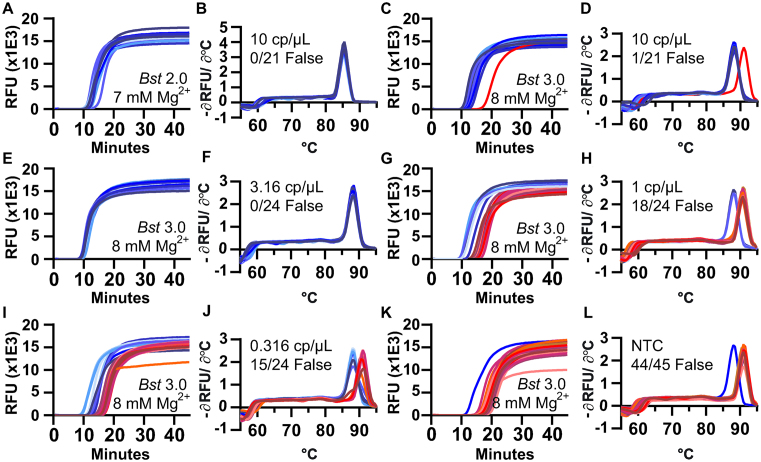
Amplification and *T*_m_ curves of *Chlamydia trachomatis* in a bulk reaction show non-specific amplification products with high *T*_m_. Plots of fluorescence as a function of time during a LAMP reaction (**A**, **C**, **E**, **G**, **I** and **K**) and the derivative plot of fluorescence as a function of temperature for the corresponding melting curves (**B**, **D**, **F**, **H**, I and **J**). Reactions using *Bst* 2.0 at 10 copies per microliter (cp/μl) (A and B), and using *Bst 3.0* at 10 cp/μl (C and D), 3.16 cp/μl (E and F), 1 cp/μl (G and H), 0.316 cp/μl (I and J), and without template (**K** and **L**). Reactions of specific amplification are different shades of blue; non-specific amplification is different shades of red. The number of false-positive reactions is reported within each panel as N/N_reaction_ False. N_Total_ for all conditions = 159 reactions.

We first wished to test whether melting temperature (*T*_m_) could be used to separate specific and non-specific amplification in a LAMP assay run in bulk. To start, we selected a concentration near the LOD where we might observe both specific and non-specific amplification. We used extracted CT genomic DNA in the presence of two commercially available polymerases, *Bst* 2.0 and *Bst* 3.0, with CT 23S as the amplification target. At target molecule concentrations of 10 copies per μL (cp/μl), amplification using *Bst* 2.0 polymerase began between 10–11 min (Figure 1A) and had uniform *T*_m_ (Figure 1B). Amplification using *Bst* 3.0 polymerase (Figure 1C), also yielded amplification from 10–11 min; however, we also observed a non-specific amplification at 15 min, defined as having a different *T*_m_ than the specific amplification events (Figure 1D). This indicated *Bst* 3.0 could be a useful model for studying non-specific amplification. We observed that early amplifying products corresponded to specific amplification events, and the later products corresponded to non-specific amplification, supporting our prediction that we could use *T*_m_ as a proxy for sequence identity, as is common with PCR and has been used previously in LAMP (25–29).

**Figure 2.**
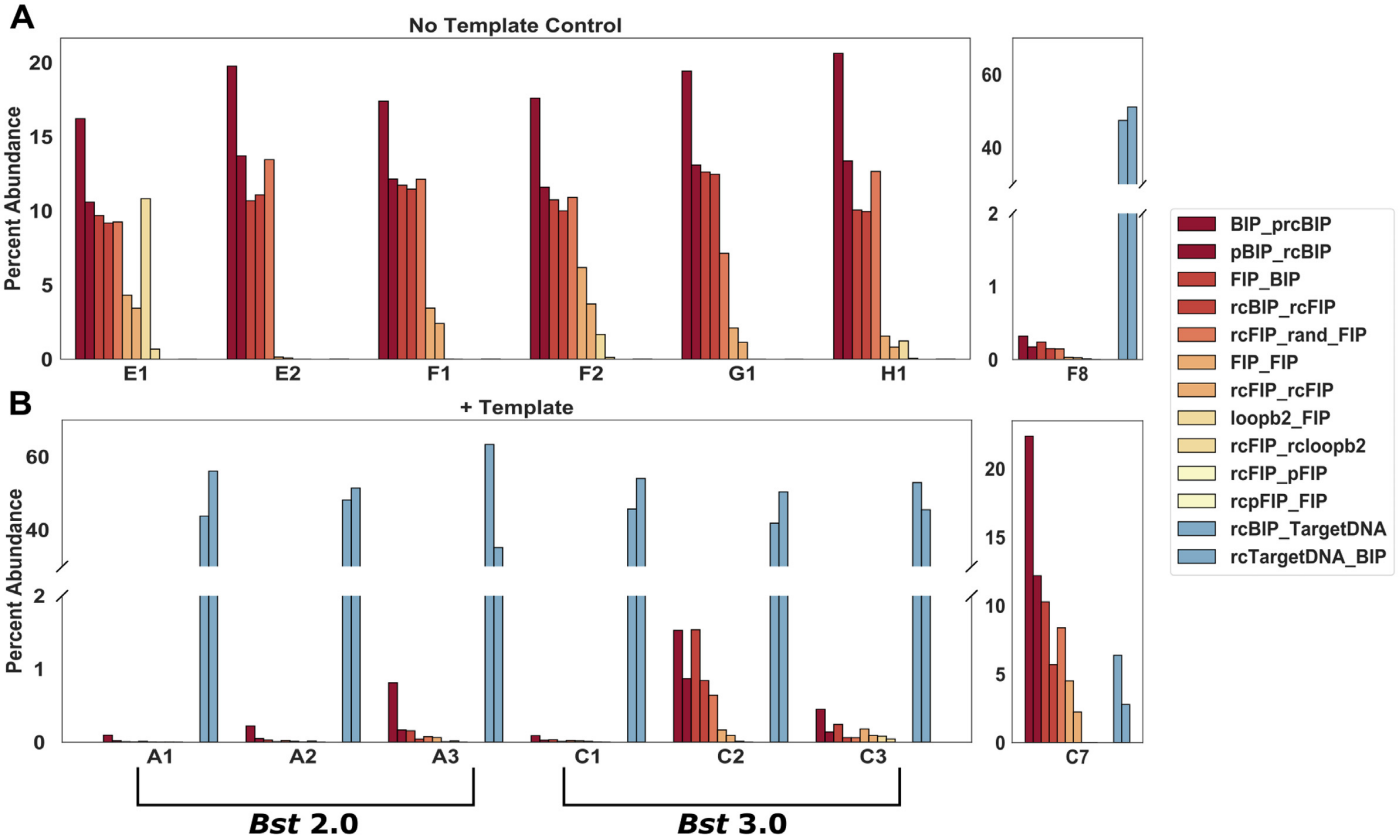
Quantification of junctions using next-generation sequencing of select *Chlamydia trachomatis* amplification products from bulk reactions. Non-specific amplification from the no-template control using *Bst* 3.0 (**A**), including amplification of a specific target contamination (well F8) corresponding to Figure 1K and L. Amplification in the presence of 10 cp/μl template (**B**), using *Bst* 2.0 (wells A1-A3) corresponding to Figure 1A and B, and *Bst* 3.0 (wells C1-C3) corresponding to Figure 1C and D. Non-specific amplification in the presence of 10 cp/μl template and *Bst* 3.0 (well C7) corresponding to Figure 1C and D. For a complete list of abbreviations used in this figure, see Supplementary Table S2.

Using *Bst* 3.0 at low concentrations of target is a useful system to study non-specific amplification. To investigate the role of the concentration of the target on the incidence of non-specific amplification, we performed half-log dilutions of template from 10 to 0.316 cp/μl. At 3.16 cp/μl (Figure 1E and F), only specific amplification occurred (24 replicate wells/plate). However, once template concentrations reached 1 cp/μl (Figure 1G and H), non-specific amplification occurred with greater frequency than specific amplification (18 of the 24 replicates generated false positives). Similarly, for 0.316 cp/μl (Figure 1I and J) 15 of the 24 replicates generated false positives. We next ran the same assay in the absence of template (no-template control, NTC) (Figure 1K and L). Even though we did not expect amplification, we observed all reactions amplified. A total of 44 of 45 replicates amplified at a *T*_m_ of 91°C, consistent with the *T*_m_ of non-specific amplification in the presence of template. Although it is possible for a reaction to generate multiple different non-specific amplification products, even ones with *T*_m_ matching to the specific products, the single amplicon observed at 88°C in the NTC was a contaminant that appeared to have the same sequence as the specific products (Figure 2A [well F8]). In general, when the specific target was present, it amplified sooner and outcompeted the non-specific amplification, thereby reducing the number of observations of non-specific amplification. To determine if the non-specific amplification was inherent to the polymerase or a consequence of buffer selection, we conducted additional studies using both *Bst* polymerases (Supplementary Figure S1 and Table S1).

**Figure 3.**
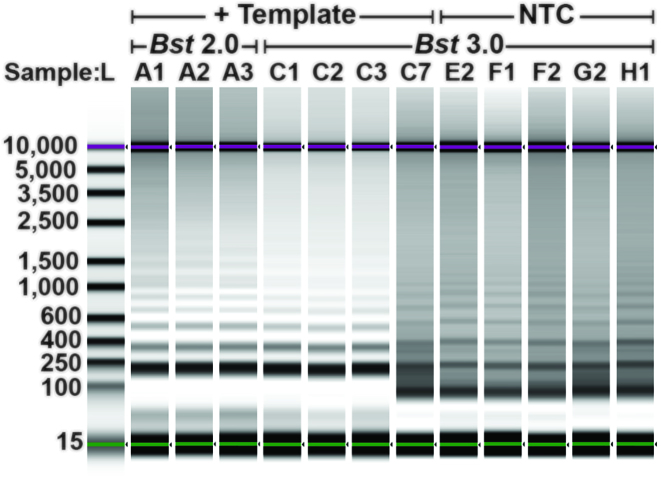
Composite image of select *Chlamydia trachomatis* amplification products from a bulk reaction. Products were collected using D5000 tape on an Agilent TapeStation. Amplification in the presence of 10 cp/μl template using *Bst* 2.0 (lanes A1-A3) corresponding to Figure 1A and B, and *Bst* 3.0 (lanes C1-C3, C7) corresponding to Figure 1C and D. Non-specific amplification in the no-template control (NTC; lanes E2-H1) correspond to Figure 1K and L. Contrast was determined using the automatic ‘scale to sample’ feature in the Agilent TapeStation analysis software.

To better understand non-specific amplification in LAMP, we investigated the sequence identity of the non-specific products with high *T*_m_ using sequencing and gel analysis and compared them with the specific products. The *T*_m_ of specific amplification differed between the two polymerases tested. Specific amplification for *Bst* 2.0 had a *T*_m_ of 85.5°C, whereas specific amplification using *Bst* 3.0 had a *T*_m_ of 88°C, and demonstrated non-specific amplification at *T*_m_ of 91°C. The non-specific amplification had identical *T*_m_ to amplification in absence of template (Figure 1K and L). Despite the specific amplification products of *Bst* 2.0 and *Bst* 3.0 producing similar gel banding patterns (Figure 3) and the same sequencing results (see Figure 2B), they had different *T*_m_ (Figure 1B and D, respectively). We determined the difference in *T*_m_ was due to differences in buffer conditions (Supplementary Figure S1 and Table S1).

In all bulk reactions, we observed non-specific products with high Tm. This was surprising because in PCR primer dimers have low Tm; moreover, in previous demonstrations of LAMP, *T*_m_ was lower for non-specific compared with specific products (27). Thus, we investigated the sequence identity of the non-specific product with high *T*_m_. We ran the LAMP products on a gel and observed that the characteristic pattern of the specific amplification products differed substantially from the banding pattern seen in the high-*T*_m_ non-specific products (Figure 3). Interestingly, the high-*T*_m_ non-specific product had a ladder pattern resembling that of specific LAMP products.

To determine the identity of the high-*T*_m_ non-specific products, we performed next generation sequencing (NGS). We observed that the non-specific products lacked the corresponding target sequence and identified the product as a mixture of full-length FIP, BIP and their complements, as well as fragments of BIP (Figure 2A).

To confirm the sequence identity of the amplicon, we targeted the FIP and BIP regions using several restriction endonucleases. Digestion of the specific and non-specific products resulted in different banding patterns than the undigested samples, and was consistent with the presence of both FIP and BIP endonuclease recognition sites within the sequence (Supplementary Figure S2). Specific amplification products were 47% GC; non-specific amplification products were 53% GC.

### A proposed mechanism for formation of non-specific product

We hypothesize a mechanism for the formation of the non-specific product with high *T*_m_ originating as a consequence of interactions of the *Bst* polymerase and LAMP inner primers. Other potential mechanisms include LIMA (30) and UIMA (31), but are inconsistent with our sequencing results, which observe nearly equal reads of the forward and reverse strand as measured by counting the complementary sequences between each junction. Our proposed mechanism requires properties that have been observed with *Bst* enzymes: a strand-displacing polymerase lacking 3′-5′ exonuclease activity—common to polymerases from thermophilic bacteria (32,33), template switching ability to allow synthesis across a discontinuous template (33), terminal transferase activity, or the ability to perform non-templated synthesis (32,34–35). Briefly, the non-specific product likely arises from extension of a low probability homo-dimerization of the Backward Inner Primer (BIP), followed by elongation across a discontinuous junction (‘template switching’) to form a double-stranded product incorporating Forward Inner Primer (FIP). Through breathing of the molecule, the 3′ of one strand may form a second hairpin and amplify. Some of these amplification events incorporate several random nucleotides via terminal nucleotidyl transferase activity resulting in a pool of hairpins with 3′ randomers. Sequences with complementary randomers are selected *in vitro* to amplify. The double-stranded product of this amplification can, through intramolecular hydrogen bonding, form two dumbbell-like structures and amplify in a fashion similar to the standard LAMP mechanism, but primed by BIP. Repetitive cycles of self-priming and hairpin priming by BIP result in numerous sequences with complementarity and the possibility of multiple replication loci within a single molecule. This process can give rise to very long amplicons, and even a branched, mesh-like network from the multimeric sequences annealing to their neighbors or in a self-complementary fashion. A simplified version of this mechanism, annotated with sequencing data, can be found in Supplementary Figure S3.

**Figure 4.**
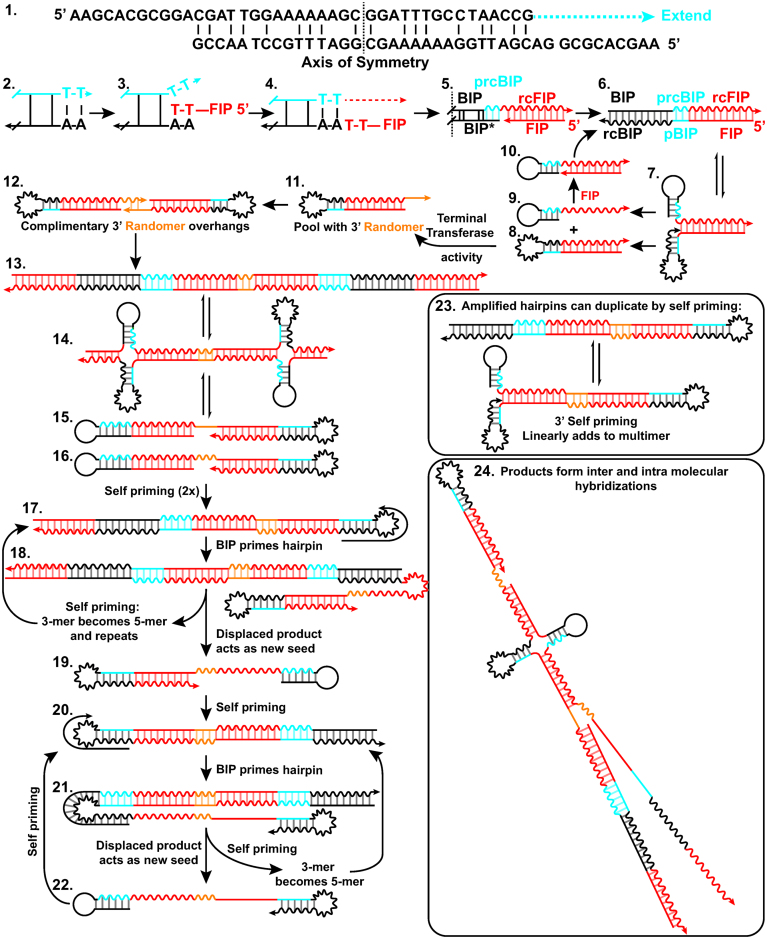
Illustration of a mechanism for formation of non-specific amplification products in LAMP reactions. Putative structures and intermediates are labeled with numbers. Forward sequences are illustrated as a straight line, and the reverse compliment as a wavy line of matching color. Abbreviations used in this figure: BIP, Backward Inner Primer; rcBIP, Reverse compliment of BIP; FIP, Forward Inner Primer; rc FIP, Reverse Compliment of FIP; prcFIP, Partial Reverse Compliment of FIP.

In more detail, a potential mechanism of formation of non-specific products is as follows: initially, a double-stranded amplicon is generated by homo-dimerization of BIP, and 3′ extension of the homodimer to produce a partial reverse complement of BIP (prcBIP) (Figure 4-1). *Bst* polymerase is highly prone to mismatched extension (36), and the two base pairs of CG provide a sufficient anchoring in the 3′ to start elongation. Multiple Primer Analyzer (ThermoFisher) does not identify the BIP homodimer, unless maximum sensitivity is used. Alternatively, BIP-prcBIP product may arise from a single stranded BIP-hairpin, as has been observed by others (37), although UNAfold (IDT) does not predict the formation of the hairpin for this primer. These structures may not need to be abundant at equilibrium, but as long as they are extended by the polymerase, the product will be stabilized and will accumulate.

**Figure 5.**
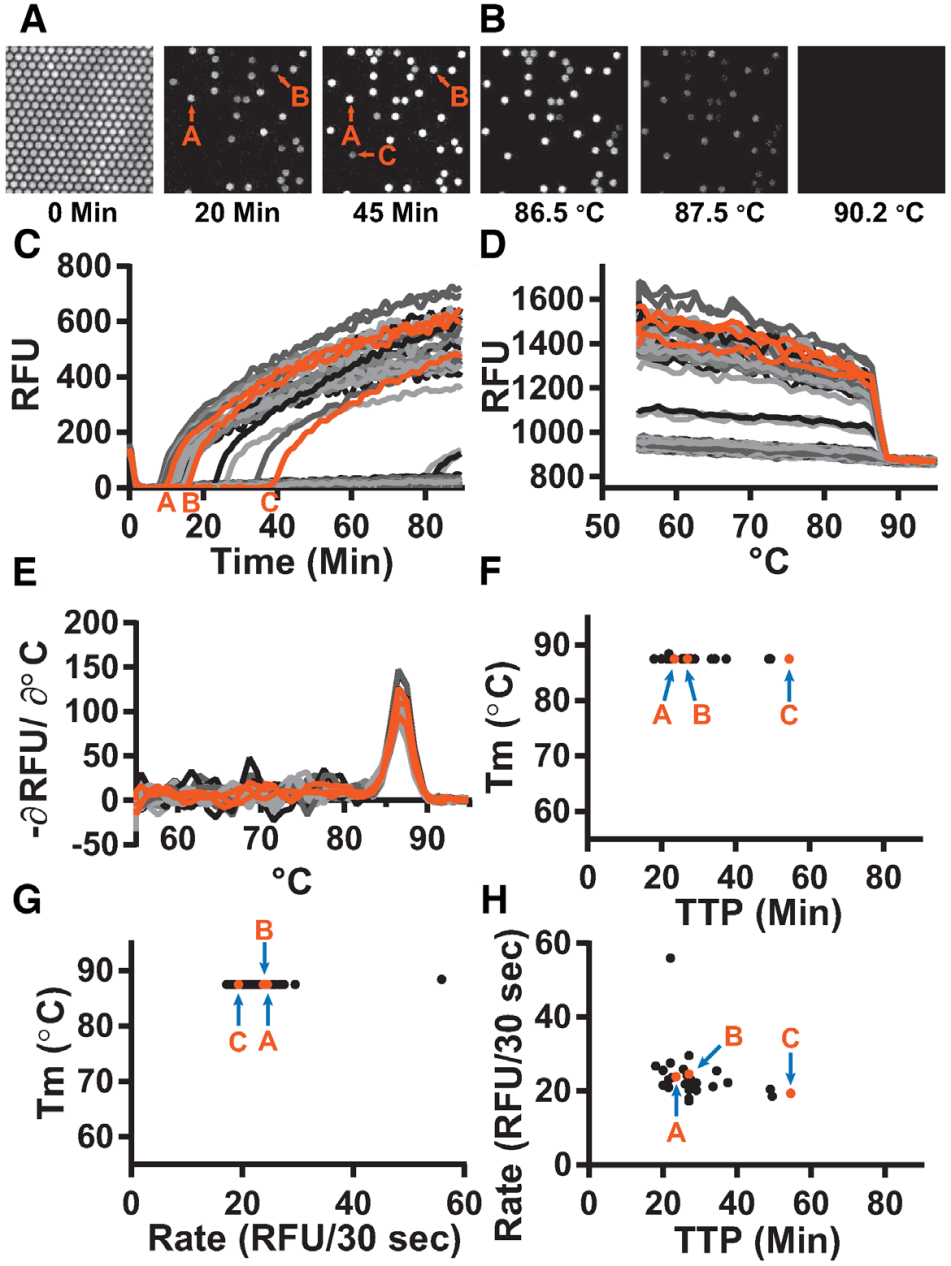
Specific amplification in digital single-molecule experiments using *Bst* 2.0. (**A**) Fluorescence micrographs of individual partitions are traced over time. For simplicity, we illustrate a subset of 250 of 20,000 possible partitions at three time points (0, 20 and 45 min). Of the 250 partitions in this micrograph, 30 partitions amplified. Partitions A and B are visible at 20 min; partition C becomes visible at 45 min. (**B**) Fluorescence micrographs of individual partitions are traced across temperatures during an HRM experiment. As the double-stranded DNA in each partition de-hybridizes, the intercalating dye is released and fluorescence decreases. (**C**) Plotting the fluorescence intensity as a function of time generates the standard amplification traces of individual partitions generated during a 90-min LAMP experiment. Orange curves correspond to partitions A–C from panel A. (**D**) Traces of fluorescence intensity as a function of temperature for individual partitions during melting experiments. By quantifying real-time intensity of individual partitions as temperature increases, melting traces are obtained. Temperature resolution is 1°C from 55–90°C, and 0.5°C from 90–95°C. (**E**) The derivative plot of panel D generates the standard melting curve. The temperature at which the derivative maximum occurs corresponds to the ‘melting point’ of the LAMP products in the individual partition. (**F**) The time each partition reached a fluorescence intensity of 250 RFU (TTP) as a function of temperature. (**G**) Maximum rate as a function of *T*_m_ for each partition. (**H**) TTP as a function of maximum rate for each partition.

**Figure 6.**
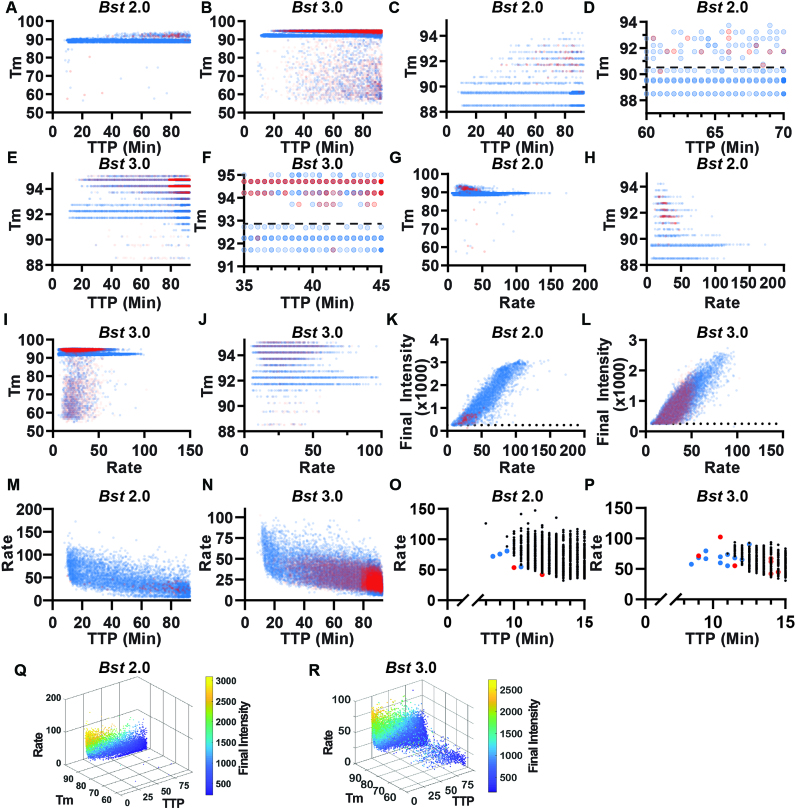
Properties of specific and non-specific amplification using real-time kinetics and *T*_m_. Blue indicates amplification events in the presence of template, red indicates amplification in the absence of template (NTC). Among these amplification events, true positives were identified using *T*_m_ (88.5–90.3°C for *Bst* 2.0 and 91.25–92.75°C using *Bst* 3.0). Color intensity indicates the abundance of paritions at a given TTP and temperature (partitions in panels **A**, **C**, **D**, **G**, **H**, **K**, **M** using *Bst* 2.0 are rendered at 20% opacity in the NTC and in the presence of template; panels **B**, **E**, **F**, **I**, **J**, **L**, **N** using *Bst* 3.0 are rendered at 5% opacity in the NTC and 20% in the presence of template. (A) *T*_m_ of individual amplification events as a function of TTP using *Bst* 2.0. (B) *T*_m_ of individual amplification events as a function of TTP using *Bst* 3.0. (C) Individual partitions with *T*_m_ between 88 and 95°C as a function of TTP using *Bst* 2.0. (D) Individual partitions with *T*_m_ between 88 and 95°C and TTP between 60 and 70 min using *Bst* 2.0. Dashed line at 90.3°C indicates the upper threshold separating specific and non-specific amplification. (E) Individual partitions with *T*_m_ between 91 and 95°C as a function of TTP using *Bst* 3.0. (F) Individual partitions with *T*_m_ between 91 and 95°C and TTP between 35 and 45 min using *Bst* 3.0. Dashed line at 92.75°C indicates the upper threshold separating specific and non-specific amplification. (G) *T*_m_ of individual amplification events as a function of maximum rate using *Bst* 2.0. (H) *T*_m_ of individual amplification events between 88 and 95°C as a function of maximum rate using *Bst* 2.0. (I) *T*_m_ of individual amplification events as a function of maximum rate using *Bst* 3.0. (J) *T*_m_ of individual amplification events between 88 and 95°C as a function of maximum rate using *Bst* 3.0. (K) The final intensity of individual amplification events as a function of maximum rate using *Bst* 2.0. (L) The final intensity of individual amplification events as a function of maximum rate using *Bst* 3.0. (K and L) Partitions with a final intensity <250 RFU (dotted line) were excluded from analyses. (M) The maximum rate of individual amplification events as a function of TTP using *Bst* 2.0 and (N) using *Bst* 3.0. (**O**) Plot of maximum rate from false-positive amplifications in NTC (red), false positives amplifications in the presence of template (blue) and true-positive amplifications by *T*_m_ (black) as a function of TTP using *Bst* 2.0 and (**P**) using *Bst* 3.0. (**Q**) 3D plots comparing maximum rate, *T*_m_, TTP and final intensity of individual partitions using *Bst* 2.0 and (**R**) using *Bst 3*.0.

**Figure 7.**
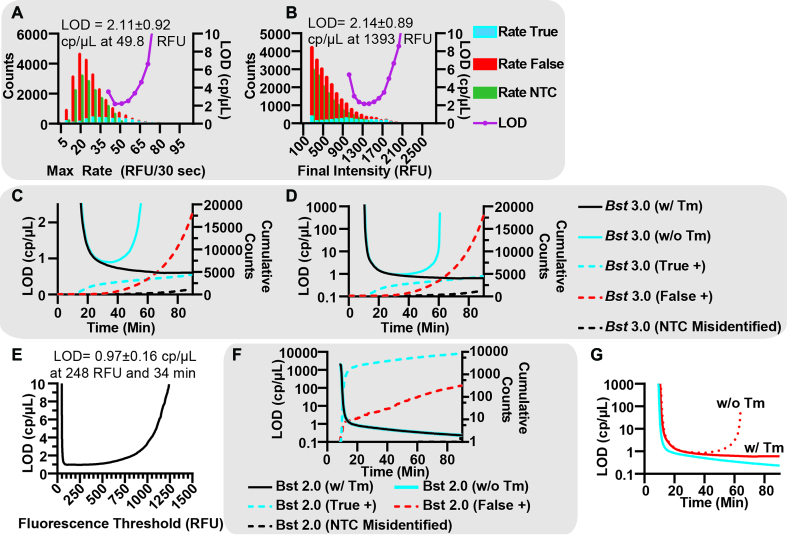
Classification of amplification reactions using HRM to determine optimal performance of dLAMP assays. (**A**) Histogram of the false positives identified by *T*_m_ within the presence of template (red), true positives by *T*_m_ (blue) and false positives in the NTC (green), binned by max rate of the partition and an LOD curve plotted as a function of max rate using *Bst* 3.0. (**B**) Histogram of the false positives identified by *T*_m_ within the presence of template (red), true positives by *T*_m_ (blue) and false positives in the NTC (green), binned by final intensity of the partition and an LOD curve plotted as a function of final intensity using *Bst* 3.0. (**C**) LOD Curves using *Bst* 3.0 as a function of time without using in the final assay (blue) and using *T*_m_ in the final device (black). Plots of cumulative counts of true positives (red dashed), false positives (blue dashed) and incorrectly identified partitions (black dashed). (**D**) Logarithmic plot of LOD curves using *Bst* 3.0 as a function of time without using *T*_m_ in the final assay (blue) and using *T*_m_ in the final device (black). Plots of cumulative counts of true positives (red dashed), false positives (blue dashed) and incorrectly identified partitions (black dashed). (**E**) LOD plotted as a function of fluorescence intensity, when the assay is measured at the optimal TTP of 34 min. (**F**) Logarithmic plot of LOD curves, using *Bst* 2.0, as a function of time without using *T*_m_ in the final assay (blue) and using *T*_m_ in the final device (black). The blue and black plots overlay. Plots of cumulative counts of true positives (blue dashed), false positives (red dashed) and incorrectly identified partitions (black dashed). (**G**) Plot of LOD curves as a function of time comparing *Bst* 2.0 (solid blue with *T*_m_, dotted blue without *T*_m_) and *Bst* 3.0 (solid red with *T*_m_, dotted red without *T*_m_). Curves for *Bst* 2.0 overlap.

**Figure 8.**
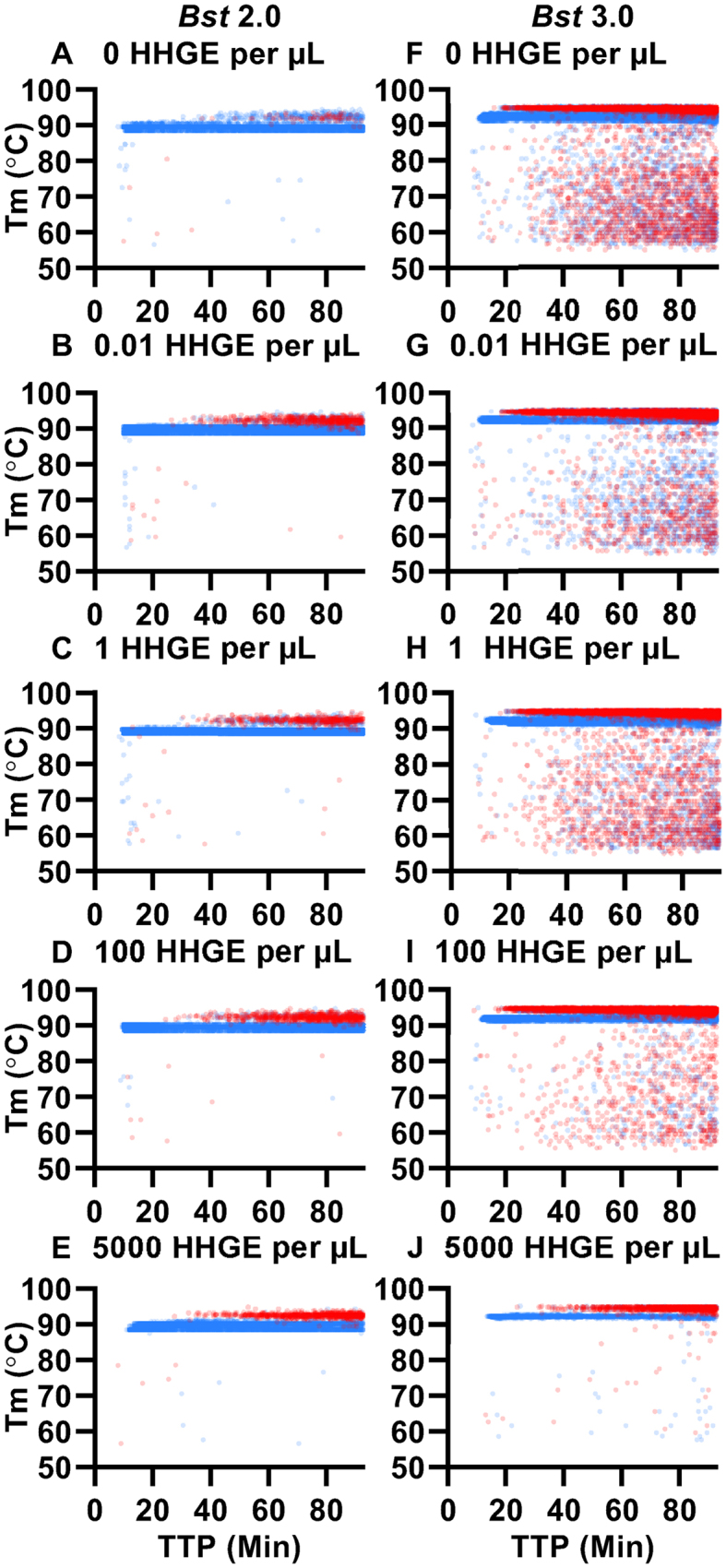
Impacts of host (human) genomic DNA in human haploid genome equivalents (HHGE) on specific and non-specific amplification. Plots of *T*_m_ as a function TTP using *Bst* 2.0 at (**A**) 0 HHGE per μl; (**B**) 0.01 HHGE per μL, (**C**) 1 HHGE per μl, (**D**) 100 HHGE per μl and (**E**) 5000 HHGE per μl; and using *Bst* 3.0 at (**F**) 0 HHGE per μl, (**G**) 0.01 HHGE per μl, (**H**) 1 HHGE per μl, (**I**) 100 HHGE per μl (**J**) 5000 HHGE per μl in the presence of template (blue) and NTC (red). *N* = 3 for all conditions, except *Bst* 3.0 at 0 and 100 HHGE per μl in the presence of template, where *N* = 6.

**Figure 9.**
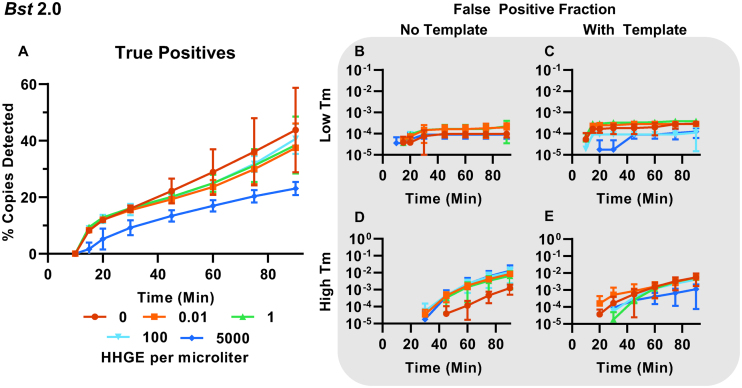
Quantification of the impact of hgDNA on specific and non-specific amplification using *Bst* 2.0 a as a function of time. (**A**) The percentage copies detected (specific amplification) as a function of time. (**B** and **C**) The fraction of partitions with non-specific amplification with *T*_m_ less than the specific amplification in the NTC (B) and in the presence of template (C) as a function of time. (**D** and**E**) The fraction of partitions with non-specific amplification with *T*_m_ greater than the specific amplification in the NTC (D) and in the presence of template (E) as a function of time. Panel (A) is available in tabular form as Supplementary Table S9.

**Figure 10.**
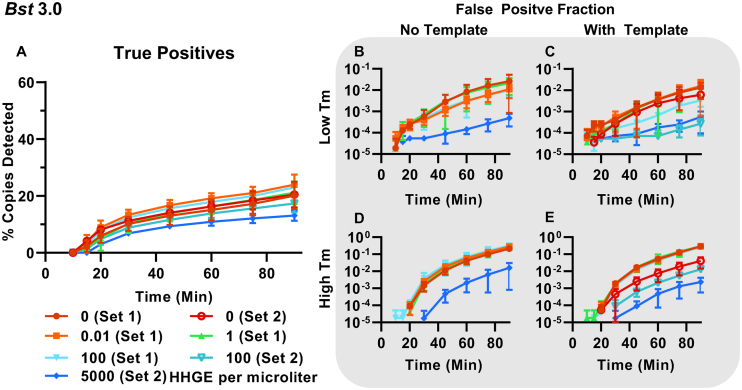
Quantification of the impact of hgDNA on specific and non-specific amplification using *Bst* 3.0 as a function of time. (**A**) The percentage copies detected (specific amplification) as a function of time. (**B** and **C**) The fraction of partitions with non-specific amplification with *T*_m_ less than the specific amplification in the NTC (B) and in the presence of template (C) as a function of time. (**D** and **E**) The fraction of partitions with non-specific amplification with *T*_m_ greater than the specific amplification in the NTC (D) and in the presence of template (E) as a function of time. Panel (A) is available in tabular form as Supplementary Table S10.

Upon accumulation of the BIP-prcBIP construct, the reverse complement of FIP (rcFIP) is incorporated by template switching (Figure 4-2). The 3′ of FIP is within spatial proximity of the homo-BIP sequence due to microhomology of to 5′ end of the double-stranded sequence coupled with rapid breathing of two base pairs of TA. This allows temporary insertion and hybridization of FIP with the double-stranded BIP-prcBIP sequence (Figure 4-3). When the polymerase is also in proximity of this reaction, FIP slips out of the junction, and the polymerase elongates across the 3′ discontinuous junction (33,35) templated by FIP (Figure 4-4). We confirmed the interaction of FIP and BIP produced the high-*T*_m_ non-specific amplification, and that elimination of 3′ microhomology could significantly reduce high-*T*_m_ non-specific amplification (Supplementary Figures S4–6 and Tables S3–4). After elongation, the FIP which has served as template, is poised to prime in the opposite direction (Figure 4-5). This either displaces the initial BIP mispairing (BIP*) or opens the hairpin, resulting in a double-stranded BIP-prcBIP-FIP product (Figure 4-6). This three part junction is observed as a complete product in NGS data. Breathing of double-stranded BIP-prcBIP-FIP is prone to formation of an intramolecular self-priming hairpin of rcBIP-pBIP (Figure 4-7). Elongation of the 3′ hairpin results in a double-stranded FIP-pBIP-rcBIP-rcFIP hairpin (Figure 4-8) and displacement of a BIP-prcBIP-rcFIP hairpin (Figure 4-9), which may be primed by FIP to restart this cycle (Figure 4-10). With each amplification, and re-prime by FIP, a single product is generated. This process of hairpin accumulation would cause the linear ‘rinsing’ baseline observed by other researchers (37).

Within this pool of linear amplifying products, the *Bst* enzyme will randomly incorporate additional nucleotides at the 3′ end of FIP-pBIP-rcBIP-rcFIP via terminal transferase activity (Figure 4-11). Our sequencing methods are unable to observe a FIP-randomer hairpin because adapter ligation requires double-stranded products. This pool of hairpins with random sequences will accumulate until LAMP selects for sequences that amplify by sharing complementary 3′ ‘toe holds’ (Figure 4-12). Much like *in vitro* evolution, those sequences with the highest probability of amplification are selected (32). The lack of a thermal gating mechanism in LAMP and lack of 3′–5′ exonuclease activity makes the amplification reaction especially prone to *in vitro* evolution of self-amplifying products. When considered in this light, it is unsurprising that non-specific amplification could arise from mechanisms similar to the specific products. Within a given bulk reaction, variation in randomer sequence length and identity was low. However, between different samples, randomer sequences of multiple lengths and identities were observed. These two results further suggest that in bulk reactions amplification occurs from one or a few sequences (Supplementary Table Groups S5–7).

Elongation from the randomer overhang results in a double-stranded products, leading to dumbbell structures, and LAMP-like amplification. First, elongation of hairpins with complementary randomer toe holds produces a dimer of FIP-BIP-prcBIP-rcFIP coupled through the randomer (Figure 4-13). Breathing of the molecule can result in formation of intramolecular hairpins, and eventual disassociation into two separate self-priming, dumbbell shaped hairpins (Figure 4-15 and -16). The products of elongation from self-priming amplification doubles the amount of dsDNA present and forms sequences with internal hairpins capable of priming by BIP (Figure 4-17). Elongation from BIP priming creates a new double-stranded product and reveals a self-priming 3′ hairpin of the original strand (Figure 4-18), which upon elongation, displaces the sequence primed by BIP (Figure 4-19) while transforming the trimer of FIP-BIP-prcBIP-rcFIP to a pentamer (more than tripling the amount of ds products from structures 15 and 16). The pentamer still contains an rcBIP hairpin, and may amplify in a functionally similar method as previously (Figure 4-17). The displaced product Figure 4-19 is similar to Figure 4-16 but missing 5′-FIP. However, similar to Figure 4-16, this product is self-priming and produces a structure with an internal rcBIP hairpin (Figure 4-20). A second priming of the hairpin by BIP of the rcBIP-pBIP hairpin and subsequent elongation, creates a new double-stranded product and reveals a self-priming 3′ hairpin of the original strand (Figure 4-21). As previously, upon elongation, the sequence primed by BIP is displaced (Figure 4-22). Simultaneously, the self-priming event turns the FIP-BIP-prcBIP trimer to a pentamer, which may continue to be amplified by BIP. The released sequence (Figure 4-22) is again self-priming, and whose product is equivalent to Figure 4-20 to restart the cycle. Further, amplified hairpins may, in addition to BIP priming of the hairpin, duplicate through self-priming by breathing and formation of a 3′ rcBIP-pBIP hairpin (Figure 4-23).

The products of these reactions are capable of forming a branched, mesh-like network resulting in the observed high temperature melting. Products may experience random internal priming by through hairpin formation (e.g. Figure 4-13,-17,-20), or 3′ self-priming (Figure 4-23). Consequently, multiple replication loci may exist within a single strand and products may have internal stem loop structures (Figure 4-24). Furthermore, in addition to intramolecular bonding, the highly repetitive nature of these products allows for melting of internal fragments, which reanneal to self in a different conformation, or a neighboring strand.

Though the initial steps of generating a double-stranded hairpin will be unique to our particular primer set, once a seed is generated, the processes of template switching and terminal transferase activity should be a general phenomenon associated with non-specific amplification of thermophilic polymerase resulting in exponential amplification. As evidence, when the mechanism of seed formation is disrupted through elimination of the microhomology, amplicons with high *T*_m_ still occur, albeit with lower frequency and delayed occurrence (Supplementary Figures S4–6 and Tables S3–4). Template switching and non-template synthesis are 100× slower than template extension (33). However, once the self-amplifying products are selected, the reaction follows standard exponential LAMP enrichment. Thus, accumulation of a sufficient pool of randomers may take time, but still result in a delayed bulk exponential amplification event. Furthermore, should a hairpin with attached randomer form, it is possible that the rising baseline, attributed to hairpin formation (37), may also by *in vitro* selection of the products, lead to and result in spontaneous exponential amplification.

### Melting temperature differentiates specific and non-specific reactions in dLAMP

To study specific and non-specific amplification events at the digital single-molecule level, we developed a new approach that enabled HRM analysis (obtaining ‘melt curves’) to be performed on each partition. We used a commercially available microfluidic chip with 20,000 partitions and a previously published open-source dLAMP method accessible to most standard laboratories (23) with the following improvements: incorporation of an off-the-shelf thermoelectric unit to both heat and cool the chips, and an enhanced MATLAB script to allow for multicolor tracking. We used the temperature-independent fluorophore ROX to track each partition's location and the dsDNA intercalating fluorophore SYTO 9 to follow amplification and hybridization status. This two-channel approach is required to follow a partition through both amplification and the entirety of the HRM when fluorescence from SYTO 9 is lost.

As an illustration of the capabilities of our approach, we first used real-time dLAMP to study the kinetic parameters of individual reactions and we used *T*_m_ to classify reaction outcome (Figure 5). Using real-time dLAMP, we followed individual partitions as they amplified as a function of time (Figure 5A) and then by temperature as they went through HRM (Figure 5B). Real-time imaging of individual partitions enables us to reconstruct the standard amplification curves of intensity for each partition as a function of time (Figure 5C), and plotting the fluorescence intensity as a function of temperature yields an HRM trace (Figure 5D); the negative derivative plot (Figure 5E) of this melt trace is the standard melt curve. Analogous to bulk measurements, the standard melt curve is used to classify reactions as specific or non-specific. We used these classifications to identify important patterns in the kinetics of each type of amplification (Figure 5F–H).

We next used real-time dLAMP with HRM to determine whether differences in time to positive (TTP) were due to a difference in amplification initiation or in rate. We expect this information would be valuable for elucidating whether the molecules that lead to bulk amplification are the ones that are first to initiate or the ones that initiate with the fastest rates. We found that TTP can be heterogeneous while *T*_m_ is constant (28.6 ± 8.9 min with 87.5 ± 0.2°C), indicating that the same product may initiate at different times (Figure 5F). This is consistent with our knowledge of the stochastic initiation of LAMP (23,38–39). Further, we observed some variability in the maximum rate despite similar *T*_m_ (23.7 ± 6.8 RFU/30 s, with 87.5 ± 0.2°C *T*_m_), which indicates the same product may amplify at different velocities (Figure 5G). In general, we observed that maximum rate often corresponded to the point when the reaction first began to amplify. By plotting rate as a function of TTP (Figure 5H) we observed little fluctuation in rate across a range of different TTPs (23.7 ± 6.8 RFU/30 s with 28.6 ± 8.9 min), indicating that the differences in TTP are mostly delays in the initiation of amplification rather than differences in the rate of amplification.

The use of real-time data revealed heterogeneity in the timing of amplification initiation and the amplification rate, but homogeneity in *T*_m_, indicating stochasticity in initiation of amplification. In some cases, outlier data points for rate occurred. To determine whether removing these outliers impacted the distribution of enzymatic rates, we performed a non-parametric test (Supplementary Figure S7) and found no significant differences in enzymatic rates when these outliers were excluded.

We next asked whether we could observe in dLAMP the same pattern of high-*T*_m_ non-specific amplification and low-*T*_m_ specific amplification that we observed in bulk. We performed dLAMP using three chips containing template, and three chips lacking template (NTC) and observed ∼55,000 partitions for each condition. Although 60 000 partitions are possible, not all partitions filled nor can all partitions be tracked for the full duration of an experiment. For the melt curve, fluorescence readings were taken at 1°C increments from 55–90°C; and at 0.5°C increments from 90–95°C to give higher resolution. Due to slight differences in the timing between the heating element and the image collection, some chips were observed at slightly different temperatures (<0.5°C).

Our approach enabled us to differentiate specific and non-specific amplification events using HRM. When using the polymerase *Bst* 2.0 and template (Figure 6A, blue points), we observed a large band of amplification in the temperature range 88.5–90.3°C, in agreement with the *T*_m_ observed when performing the reaction in bulk (Figure 1). In contrast, the NTC (Figure 6A, red points) had very few amplification events in that temperature range (68 out of 51 279 partitions). Hence, we defined events that occurred in the *T*_m_ range 88.5–90.3°C as true positives (specific amplification events) and we defined those that occurred outside this range (in both the NTC and in the presence of template) as false positives (non-specific amplification events). When using the polymerase *Bst* 3.0, we observed a large band of amplification from 91.25 to 92.75°C in the presence of template (Figure 6B, blue points) that did not correspond with amplification in the NTC (Figure 6B, red points) so we defined these as specific amplification events. As with bulk measurements, we determined the difference in *T*_m_ between specific amplification events between *Bst* 2.0 and *Bst* 3.0 was due to the difference in buffer composition (Supplementary Figure S1 and Table S1).

During these experiments, we observed two common patterns. First, the *T*_m_ for specific amplification events was 3–5°C lower in digital compared with bulk measurements. We attribute this difference to temperature calibration; the thermocycler is calibrated to the liquid temperature, whereas the thermoelectric element measures the temperature of the heating element. Second, false positives in the NTC had predominantly high *T*_m_, which we attribute to the non-specific product we identified in the bulk reactions. We also observed differences in total amplification events between the two polymerases. Assays with *Bst* 3.0 resulted in substantially more non-specific amplification than those with *Bst* 2.0 and confirmed this was not an issue with buffer selection (Supplementary Figure S1 and Table S1). After 90 min, *Bst* 3.0 yielded 15 200 non-specific events (out of 54 337 observed paritions) in the NTC, whereas *Bst* 2.0 yielded only 74 non-specific events (out of 51 279) in the NTC. Occasionally, outliers occurred in the NTC and would be misidentified as positives by fluorescence and *T*_m_. For *Bst* 3.0 this occurred in 29 partitions; for *Bst* 2.0, it occurred in only 3 out of ∼55,000 partitions.

Next, we tested whether TTP is different for specific and non-specific amplification. Because LAMP follows a ‘winner-takes-all’ format, frequent and early non-specific amplification events may dominate bulk amplification. In general, for both *Bst* 2.0 and *Bst* 3.0, specific amplification had earlier TTP than non-specific amplification, although there was some overlap, mostly >90.5°C (Figure 6A and B). We were able to distinguish the clustering of high-*T*_m_ non-specific products separately from specific amplification using a threshold of 88.5–90.3°C (Figure 6C and Supplementary Figure S8A). We illustrate each partition with only partial opacity so that when false positives in the NTC (red) overlap with false positives in the template-containing sample (blue), the overlap of multiple colors appears purple (Figure 6D). Color intensity indicates the abundance of paritions at a given TTP and temperature. To further illustrate how this approach can be used to differentiate specific and non-specific amplification, we next selected a region where both specific and non-specific products were observed. For *Bst* 3.0, we were able to distinguish the clustering of high-*T*_m_ non-specific products separately from specific amplification using the threshold of 91.25–92.75°C (Figure 6E) and we observed better separation of specific and non-specific amplification than with *Bst* 2.0 (Figure 6F and Supplementary Figure S8B). Both enzymes had highly variable TTP, which we have observed previously (23) and attribute to stochastic initiation of LAMP. *Bst* 2.0 had both earlier specific amplification and later non-specific amplification than *Bst* 3.0. *Bst* 2.0 reactions containing template generally started at 10 min, whereas non-specific amplification began at ∼40 min. In contrast, *Bst* 3.0 reactions containing template began at 11.5 min and non-specific amplification began at ∼20 min.

Next we asked whether there is a difference between the maximum rates of specific and non-specific amplification. Previously, we demonstrated that rate could be used to correct for some non-specific amplification using *Escherichia coli* 23S primers (23), so we wished to test whether we could use maximum rate as a way to differentiate specific and non-specific amplification. Generally, specific and non-specific amplification reactions did not have the same maximum rate. For *Bst* 2.0, non-specific amplification tended to have a slower max rate than specific amplification, although there was some overlap (Figure 6G). At high *T*_m_, the clustering of non-specific amplification in both the presence of template and in the NTC were observed at >90.5°C and below ∼50 RFU/30 s (Figure 6H). For *Bst* 3.0, although there was substantial overlap, we again observed that non-specific amplification tended to have slower maximum rate than specific amplification (Figure 6I). Examining the high-*T*_m_ amplification events, non-specific amplification collects above 92.75°C and has maximum rate extending out to 75 RFU/30 s (Figure 6J). For both enzymes, overlap between specific and non-specific amplification was similar and specific amplification tended to be faster. However, the maximum rate of specific amplification between the two enzymes differed; *Bst* 2.0 had a maxium rate of 150 RFU/30 s, whereas *Bst* 3.0 did not exceed 100 RFU/30 s. *Bst* 2.0 performing faster than *Bst* 3.0 is consistent with our previous observations using an *E. Coli* 23S primer set (23). Additionally, the maximum rate of non-specific amplification in *Bst* 2.0 tended to be lower than non-specific amplification in *Bst* 3.0 (50 and 75 RFU/30 s, respectively). Consequently, the extent of overlap of specific and non-specific amplificaiton was greater for *Bst* 3.0 than *Bst* 2.0.

We observed an unexpected relationship between the final intensity of each partition and the maximum rate of that partition. After 90 min of amplification, a partition should theoretically reach a fluorescence maximum whereby all reagents are consumed, amplification plateaus and thus the final intensity would be independent of the maximum rate of amplification. However, surprisingly, we observed a general scaling between the maximum rate and the final intensity of the partition. For *Bst* 2.0, all amplification in the NTC has final intensity <1017 RFU and maximum rate <53.4 RFU/30 s. In the presence of template, 79.7% of non-specific amplification and 52.3% of specific amplification had final intensity and maximum rate less than these thresholds. For *Bst* 3.0, 87.7% of amplification in the NTC has final intensity <1017 RFU and maximum rate <53.4 RFU/30 s. In the presence of template, 89.0% of non-specific amplification but only 45.6% of specific amplification fell within these thresholds using *Bst* 3.0. Thus, false positives were generally dimmer and had slower maximum rates than most true‐positive events. When examining the brightest partitions, *Bst* 2.0 (Figure 6K) and *Bst* 3.0 (Figure 6L) exhibit a similar maximal final intensity near 3000 RFU. These maxima are also surprising, considering our 12-bit camera is capable of imaging up to 4096 RFU (the detector was not at saturation). We suspect that this maxima corresponds to consumption of one of the reagents; while scaling between maximum rate and final intensity occurs when stochastically initiated reactions have not completely amplified, resulting in partitions dimmer than the maxima and proportional to their rate of amplification.

During these dLAMP experiments, we also observed a relationship between maximum rate and TTP. In bulk reactions, the first and fastest amplification event determines the reaction outcome by consuming all of the reagents. Thus, we hypothesized that reaction conditions that promote fast and early amplification in the NTC would lead to a high false-positive rate in bulk and thus misidentification of amplification. In both *Bst* 2.0 (Figure 6M) and *Bst* 3.0 (Figure 6N) we observed a general trend of fast amplification events occurring earlier, and slow events occurring later. In *Bst* 2.0, we observed greater heterogeneity in TTP and rate than in *Bst* 3.0. Furthermore, non-specific amplicons in the NTC tended to produce slower and later amplification events. Occasional outliers occurred at both fast and early times.

Next, to explicitly test whether fast and early events correspond to specific amplification, we analyzed the relationship between a partition's TTP, its maximum rate, and *T*_m_. In the first 12 min of amplification, we observed six non-specific amplification events in *Bst* 2.0 (four in the presence of template; two in the NTC; Figure 6O), and we observed 13 non-specific events in *Bst* 3.0 (10 in the presence of template; three in the NTC; Figure 6P). For both polymerases, we were able to distinguish the rare, fast and early non-specific amplicons from true positives. For *Bst* 2.0, these non-specific amplifications were slower than the fastest true positives, and occurred at similar times. In contrast, for *Bst* 3.0, the earliest amplification events were false positives and tended to have similar rates to the true positives. We hypothesize that in bulk reactions, the fast and early non-specific amplification events (as seen in *Bst* 3.0 Figure 6P) lead to non-specific measurements, whereas non-specific amplification that coincides with specific amplification, but proceeds at a slower rate (as seen in *Bst* 2.0 Figure 6O), would still produce specific amplification in bulk. This hypothesis is corroborated by sequencing of bulk LAMP reactions (Figure 2). Though individual bulk reactions may be assigned a homogeneous label as ‘true positive’ or ‘false positive’ by *T*_m_, sequencing identifies multiple products within each reaction and the *T*_m_ is determined by the dominant product. For example, we observed a ‘false positive’ by *T*_m_ (Figure 1C and D), despite the presence of template. The sequencing of this product, contained non-specific product sequences, similar to those observed in the NTC, at high prevalence, as well as the specific target sequences in low abundance (Figure 2 [well C7]). Similarly, though ‘true positive’ is assigned to other bulk reactions in the presence of template, the non-specific products are still observed at low abundance (e.g. Figure 2 [well F8]). Further, a greater number of non-specific partitions in digital using *Bst* 3.0 than *Bst* 2.0, is correlated with a greater number of non-specific reads despite the presence of template in the sequencing data (Comparing Figures 6A-B and 2B group A versus C). We hypothesize that the combination of real-time parameters (such as rate and TTP), combined with the ability of digital assays to yield probabilities and to assign reaction identity through HRM, may ultimately help researchers optimize bulk reaction conditions.

### A complex interplay exists among TTP, max rate, final intensity and *T*_m_

To better visualize how TTP, max rate, final intensity and *T*_m_ data are interrelated, we next plotted these data in a four-dimensional (4D) space (Figure 6Q-R, Supplementary Videos S1 and 2). We observed that among all partitions, regarless of if the product was specific or non-specific amplificiation, fluorescence was brighter when amplification occurred earlier and faster. This was true for both polymerases. Additionally, we observed two types of non-specific amplification. The first type of non-specific was the traditional ‘primer-dimer’ cloud, which is characterized by a low *T*_m_, low final fluorescence intensity, a slow max rate and a generally late TTP. The second type of non-specific cloud matches only in its high *T*_m_, and spans a wide range of rates, TTP and final intensities. The high-*T*_m_ non-specific amplification occurs with greater frequency than the low-*T*_m_ non-specific amplification. The major differences between the polymerases can also be resolved with this visualization. The number of non-specific amplification events is much fewer for *Bst* 2.0 than for *Bst* 3.0. Further, these non-specific events in *Bst* 2.0 never achieve same fluorescene intensity or maximum rate as with *Bst* 3.0. We include the 4D representation as part of our MATLAB code, and as videos in the Supplementary Data.

### Classification of true or false positives enables optimal analysis parameter selection

We next asked whether using a combination of digital real-time parameters, in conjunction with *T*_m_, could be used to improve the performance (LOD) of a dLAMP assay. For any given assay, there is a large combination of possible parameters (e.g. amplification rate, TTP, fluorescence intensity) that are used to determine when a digital partition is ‘on’ or ‘off.’ Use of these parameters and selection of thresholds will influence assay performance (analytical specificity and sensitivity). Assay performance is affected by amplification time and the combination of choices of parameters used to process the data impacting LOD, the probability of detecting a molecule (efficiency), and the clinical sensitivity and specificity. Having established that there is a direct relationship between *T*_m_, sequence identity and structure, we determined that *T*_m_ allows us to explicitly differentiate specific and non-specific amplification in dLAMP, and thus, differentiate true from false positives.

We foresee two separate situations of dLAMP analysis using HRM. First, where HRM is not incorporated in the final assay, but is used during assay development. Second, the ideal situation for quantitative performance, where HRM is incorporated into the final LAMP assay. We expect the first group of LAMP assays to exist because collecting *T*_m_ data adds additional time to an assay and requires more advanced hardware to run. This may be unideal in situations requiring more rapid diagnostics or limited-resource and field settings where the hardware may be impractical. Nonetheless, running HRM is still useful during LAMP assay development to select the optimal combination of parameters for end-point or real-time LAMP without using *T*_m_. Hence, *T*_m_ allows one to identify the correct combination of assay parameters, and how to analyze the data for best LOD.

LOD is a key parameter when optimizing clinical assays because pathogen load is low in many infections (e.g. in blood infections or asymptomatic sexually transmitted infections). We thus illustrated the optimization of parameters using improved LOD as the selection criteria. The combination of real-time dLAMP with HRM can uniquely define LOD because of the combination of digital and *T*_m_. Unlike bulk assays, which require a concentration titration curve (and are thus dependent on integrated signal intensity and enzymatic turnover), digital assays only require that an event (target molecule) is or is not observed and can be counted relative to the partition volume (40,41). The minimum LOD for any digital assay corresponds to one target or amplification event per partition volume. Hence, we can define LOD from a single concentration point by Equation (1):

(1) $$\begin{equation*}{\rm LOD} = \frac{{{C_{{\rm True}}}}}{{[{N_{{\rm True}}} - \left( {{N_{{\rm False}}} + 3\ \times \sqrt {{N_{{\rm False}}}} } \right)]/{N_{CI}}}}\ \end{equation*}$$

where *C*_True_ is the concentration of target molecules loaded by ddPCR counts in copies per microliter, *N*_True_ is the number of true positive (specific) amplification events observed on a chip, *N*_False_ is the number of non-specific amplification events observed on a chip and *N*_CI_ is the number of expected molecules for a given confidence interval. In this equation, the *N*_True_ and *N*_False_ are chip-specific, and take into account the total volume of the chip, the number of partitions and the volume of partitions. Furthermore, in Equation (1), amplification efficiency is implicitly taken into account via the *N_True_* parameter (in other words, for a less efficient amplification process, a given *C*_True_ on a given chip would lead to a lower value of *N*_True_). For simplicity, Equation (1) makes the assumption that the measurements are performed at sufficiently low concentrations (as is typical for LOD experiments) that only a very small fraction of occupied partitions contain more than one molecule and therefore there is a linear relationship between *C*_True_ and *N*_True_.

The concentration loaded, *C*_True_, generates N total counts of both true- and false-positive events. We can divide this concentration by the minimal number of counts needed to identify a specific amplification event and define this as the LOD. The minimum number of counts needed to guarantee a specific amplification event is observed is determined by *N*_True_, *N*_False_ and *N*_CI_. *N*_True_ and *N*_False_ are determined empirically, whereas *N*_CI_ is calculated from the desired expected number of molecules that will yield at least one detection event for a given confidence interval (*N*_CI_) from the Poisson equation. If we require a 95% CI to observe a true positive across an entire chip, the minimum number of counted events is 3 (i.e. 5% of the time, the Poisson expected loading of three target molecules will still measure zero events.) For a 98% CI, *N*_CI_ would be four counts. Hence, all true-positive counts in excess of *N*_CI_ are counts observed above the LOD. Uncertainty in the LOD is given by Supplementary Equations S1–2.

Counting only true positives does not account for interference from false positives. In order to meet our minimum counts for detection, our equation must remove false counts (*N*_False_). The generally accepted procedure for LOD calculations with a 99.7% CI is to assign *N*_True_ only when the counts exceed the background plus three standard deviations of the background (${N_{False}} + 3\ \times \sqrt {{N_{False}}}$). We approximate the variance in the background using the counting error as three times the square root of the number of false-positive events counted and subtract those counts from the true-positive counts to yield the equation.

Using this calculation of LOD to optimize an assay has three limitations. First, Equation (1) fails to produce a number with physical meaning when the number of true-positive events (*N*_True_) is less than the number of false-positive events plus three times the standard deviation in false amplification (${N_{{\rm False}}} + 3\ \times \sqrt {{N_{{\rm False}}}}$). In this case, it is not possible to conclusively observe a true positive, and the LOD becomes irrelevant. Second, Equation (1) gives an absolute LOD. The numerator (concentration of template molecules loaded on the chip, as determined by PCR) is corrected for the probability of observing a molecule amplify (efficiency) by the true-positive counts. *N*_False_ accounts for the non-specific amplification, and *N*_CI_ accounts for the Poisson probability associated with loading a target molecule. Third, this equation is specific to digital assays.

We first sought to demonstrate the selection of optimal parameters for situations where HRM is not incorporated into the final assay. Using this process, one can pick any threshold and use *T*_m_ to determine the optimal trade-off between true and false positives. All initial experiments testing the utility of LOD, juxtaposed against receiver operating characteristic (ROC) curves, to identify optimal parameters were done using *Bst* 3.0. We began by determining the optimal thresholds for max rate, Fluorescence Intensity, and amplification time. We demonstrate optimization of all three parameters, using *T*_m_ as the arbiter, to illustrate the utility of our method.

We tested the use of ROC curves (commonly used to indicate clinical sensitivity and specificity) to compare the performance in response to a given parameter. ROC curves provide a visual representation of the ability to distinguish between a true-positive and false-positive event, as a function of a given threshold, but can be difficult to use for optimal selection of LOD. ROC curves show the fractions of true and false positives, where the true-positive fraction is the number of true positives at a given threshold out of the total number of true positives observed by *T*_m_; and the false-positive fraction is the number of false positives counted at the given threshold, divided by the total number of false positives observed by *T*_m_. A perfect classifying test will yield the largest true-positive fraction and smallest false-positive fraction.

When plotting the ROC curve for maximum rate (Supplementary Figure S9A), we observed that rate initially performs very well for eliminating false positives (the false-positive fraction is very small for very high rates). However, as the digital threshold (analogous to ROC ‘cut-point’) for rate decreases, a greater number of both false and true-positive values are counted. Closer examination of this range of thresholds (Supplementary Figure S9B) emphasizes the Youden Index at 34.6 true-positive fraction and 4.6 false-positive fraction as a possible choice for optimum threshold, although the assay performance in terms of LOD is unclear. The choice for optimal final-intensity threshold is even less clear with the ROC curve (Supplementary Figure S9C), as the ROC curves do not give clear indication of the optimal LOD (the ROC curve is a gentle concave slope). Even relatively high fluorescence thresholds do not give indications of the optimal cut-point (Supplementary Figure S9D).

Filtering using LOD revealed a clear optimum. We plot the total number of events for both true and false positives and LOD as a function of maximum rate (Figure 7A). The LOD curve revealed a clear minima, corresponding to the optimal cut-point using rate. Selecting the threshold of 49.8 RFU/30 s generated an LOD of 2.11 ± 0.92 cp/μl. Similarly, plotting LOD against final intensity resulted in a clear minima, despite the histogram appearing as a continuum and the cut-point being thus ambiguous (Figure 7B). Using final intensity, an LOD of 2.14 ± 0.89 cp/μl can be achieved at 1393 RFU.

The ROC curve for TTP presented a narrow range of thresholds, with ∼50% true-positive fraction and 2% false-positive fraction, although the precise optimal threshold was not obvious (Supplementary Figure S9E). To refine this threshold, we plotted the LOD and the cumulative counts as a function of time in both linear (Figure 7C) and logarithmic scales (Figure 7D).

Assays employing HRM only during the development of the assay can improve the LOD of the final assays by selecting (making an informed choice of the optimum threshold). The LOD decreases (blue curve) as the true positives begin to amplify (blue dashed) and increases, as the false positives amplify (red dashed). The minima for this system occurs at 34 min and 0.93 ± 16 cp/μl, striking a balance between allowing many true positives to amplify and only a small amount of false positives to occur (53.6% true-positive fraction and 1.5% false-positive fraction) and is clearly defined using the linear scale (Figure 7C). Plotting of LOD on the logarithmic scale (Figure 7D) emphasizes improperly selecting a threshold can result in several orders of magnitude loss in assay performance (for example, stopping the assay too early or allowing the assay to run for too long). Although dLAMP is robust to perturbations, selecting the appropriate duration for amplification is important.

In contrast, assays using HRM as part of the final readout can distinguish false positives from the true positives and improve LOD further by excluding non-specific amplification from the analysis. In some instances, an NTC may incorrectly identify partitions as true positives by *T*_m_ (black dashed). We incorporate these events as non-specific amplification in the case HRM is used in the final readout. If non-specific amplification is eliminated, the assay LOD (Figure 7C and F, black solid) continues to improve with time, and is only dependent on the stochastic probability that a true positive will initiate and amplify. In this scenario, there is no penalty allowing the assay to amplify for extended periods of time.

In this scenario the LOD equation simplifies to

(2) $$\begin{equation*}{\rm LOD }= \frac{{{C_{{\rm True}}}}}{{{N_{{\rm True}}}/{N_{CI}}}} = \sim\frac{{{N_{{\rm CI}}}}}{{{\rm Fraction\ copies\ detected}}}\end{equation*}$$

Additionally, there is no limitation on the number of parameters that can be used to identify the optimal LOD. Using multiple parameters to filter the data may be useful for individuals not employing HRM in the final assay or in assays only employing end-point measurement (e.g. an assay without real-time measurements will be unable to generate data on rate, but still benefit from selecting optimal assay time and fluorescence threshold). As a demonstration, we filtered first by optimal TTP, then for the optima of a second parameter. In this case, we selected the optimal TTP of 34 min, and scanned for optimal fluorescence threshold. We plotted LOD as a function of fluorescence threshold and determined that the optimal fluorescence threshold at 34 min would be 248 RFU and correspond to an LOD of 0.97 ± 0.16 cp/μl (Figure 7E).

Do filter parameters exhibit the same LOD minima when using *Bst* 2.0, as they did for *Bst* 3.0? *Bst* 2.0 had much lower non-specific background than *Bst* 3.0, and could behave similarly or may behave differently.

First, does the ROC curve for TTP display a clear optimum? Similar to the TTP ROC for *Bst* 3.0 (Supplementary Figure S9E), the TTP ROC for *Bst* 2.0 has a concave slope making choice of the optimum a matter of computation (Supplementary Figure S9F). We can visually estimate the balance of true and false-positive fraction in the range of 50% true and 10% false. Similar curves for max rate and final intensity could be generated but are not shown here.

Second, is there an advantage to using HRM in the final assay with *Bst* 2.0? To answer this question, we plot LOD and the cumulative counts of true and false positives as a function of time for *Bst* 2.0 (Figure 7F). Similarly to *Bst* 3.0, we observe LOD improve rapidly as true-positive events are counted. However, unlike *Bst* 3.0, the non-specific amplification events are few and their presence does not have an impact on LOD. Thus, when using *Bst* 2.0, the curves representing LOD with or without HRM in the final assay overlay and indicate using HRM in the final assay has no additional benefit. Furthermore, the continuously decreasing LOD with time for either case indicates that use of ROC curves to determine an optimum can be misleading. While the ROC implies that an optimum exists, the false-positive incidence is rare enough that a TTP optimum selected by LOD does not exist. Hence, assay developers may select assay time based on requirements other than LOD.

We next assessed whether we could use HRM to compare the performance of the two polymerases, to see which one would give the best LOD and which combination of hardware components would give the optimum assay performance. (Figure 7G) For both polymerases we observed a similar, rapid decrease in LOD in the initial moments as true-positive events are detected. However, we also noticed several differences. *Bst* 2.0 has a lower LOD than *Bst* 3.0 at any amplification time. We attribute this difference to the higher incidence of false positives when using *Bst* 3.0 compared with *Bst* 2.0. An additional consequence of the low false-positive incidence using *Bst* 2.0, regardless of the use of HRM in the assay, is the LOD continues to improve with time as additional true positives are counted. In contrast, *Bst* 3.0 benefits greatly from use of HRM in the final assay. If HRM is not included in the assay (Figure 7G, red dashed), a clear optimum for LOD occurs at 34 min and 0.93±0.16 cp/μL. However, if HRM is employed in the assay, the LOD more closely resembles the LOD curve for *Bst* 2.0 and improves with increased detection of true-positive events.

We made several overarching conclusions regarding improving the LOD of dLAMP using a combination of digital real-time parameters and *T*_m_. First, filter parameters can be used singly or in combination to improve the performance (LOD) of dLAMP. In certain assays one parameter may perform better than another for this selection. For this primer set, LOD for *Bst* 3.0 was lower (better) when using TTP (0.93±0.16 cp/μL) than max rate (2.11±0.92 cp/μL) or final intensity (2.14±0.89 cp/μL). Second, incorporation of HRM into the final assay readout will benefit some assays more than others. We observed incorporation of HRM as a part of the final assay improved the perofmance of *Bst* 3.0 greater than the perofmance of *Bst* 2.0, and was vital for long assay times.

### Classification demonstrates host genomic DNA alters specific and non-specific amplification in dLAMP

Assays with high clinical sensitivity and specificity are critically needed. Clinical samples of CT, originating from urine and swabs, pose an intrinsic challenge because they contain variable levels of host DNA, and DNA from other flora. The analysis of these clinical samples, needs not only to be sensitive (good LOD), but also able to function in the presence of non-specific, potentially amplifiable genomic secondary structures and other possible environmental contaminants, while remaining consistent between samples.

We sought to investigate the impact of host human genomic DNA (hgDNA) on non-specific background amplification. We hypothesized that non-specific structures (like hairpins and regulatory elements), may amplify in the presence of LAMP and contribute to non-specific background amplification. We titrated sheared buffy coat gDNA (i.e. leukocytes) concentrations from zero to 2.5 × 10^3^ cells per μl, a concentration 2.5× greater than that expected to cause interference (8) and observed the impact on specific and non-specific amplification of CT (Figure 8). We measured the concentration of hgDNA in Human Haploid Genome Equivalents (HHGE) or half the total amount of hgDNA in a diploid cell. For each concentration of host DNA and enzyme, we ran at least three chips in the presence of CT template and three in the absence of template and across multiple days and sample lots. In total, we observed 1 196 038 different reaction partitions. At the highest concentration of hgDNA, there was 3 030 000 times more hgDNA than bacterial DNA by mass.

We first asked how background DNA impacted TTP qualitatively. We observed for both *Bst* 2.0 and *Bst* 3.0 enzymes, specific and non-specific amplification were qualitative similar independent of background DNA concentration below 5000 HHGE per μl. As with previous measurements, *Bst* 2.0 rarely produced low-*T*_m_ non-specific events; whereas *Bst* 3.0 produced both high- and low-*T*_m_ non-specific events. Further, there were more non-specific amplification events for *Bst* 3.0 than *Bst* 2.0 at both high and low *T*_m_.

We next asked how background hgDNA impacts specific and non-specific amplification quantitatively. We categorized amplification events as specific and non-specific based on *T*_m_ as previously. First, we asked: Is there a relationship between fraction of template molecules amplified in dLAMP and amplification time? We then determined the total number of template copies loaded into a chip relative to the copies measured by ddPCR. If amplification initiation is stochastic, as observed in Figures 5F and 6A-B, does longer assay time increase ‘efficiency’ and thereby improve LOD when using *T*_m_ (as seen in Figure 7C and F)? We observe that for *Bst* 2.0 a large number of partitions amplify at in the first 11.5 min, followed by a second phase after 20 min where additional partitions amplify with lower frequency (Figure 9A). The mode TTP for concentrations less than 5000 HHGE per μl was ∼11.6 ± 0.2 min (Supplementary Table S8 and Figures S10A-11C). After the mode TTP, the frequency of observing specific amplification in the absence of HHGE decreases from a maximum frequency of 1.2 ± 0.1% copies detected per 30 s to a lower average frequency of 0.23 ± 0.04% copies per 30 s from 20 to 90 min (Figure 9A). For *Bst* 3.0 (Figure 10A), we observe a similar trend temporally, though mode TTP was at least 2 min slower and had greater variability than *Bst* 2.0 (Supplementary Table S8, Figures S10B-11D). Further, *Bst* 3.0 consistently amplified fewer target molecules than *Bst* 2.0 at all time points. This highlights the stochastic nature of amplification using LAMP, and importance in choice of enzyme on sensitivity. In theory, assays employing *T*_m_ could be run until all partitions amplify as either a false or true positive. Allow all partitions to amplify would give the highest possible number of target copies amplified and lowest possible LOD when using *T*_m_ in the final assay.

Second, we asked what is the impact of hgDNA on efficiency as a function of time? For both *Bst* 2.0 and 3.0 (Figures 9A and 10A), when comparing within a given enzyme, we observed that the fraction of copies detected and the moment the majority of reactions initiate, were indistinguishable for concentrations less than 5000 HHGE per μl. At 5000 HHGE per μl, a decrease in the fraction of copies detected and a delay in amplification initiation was observed (see also Supplementary Figure S11C and D). *Bst* 2.0 had a mode TTP of delay of 4.7 min to 16.3 ± 2.7 min, whereas in *Bst* 3.0, the mode TTP was 17.2 ± 2.1 min at 5000 HHGE per μl (Supplementary Table S8 and Figure S11). Thus, high concentrations of hgDNA may suppress specific amplification.

Third, we asked what is the impact of hgDNA and time on non-specific amplification? For *Bst* 2.0, we observed consistent non-specific amplification products with high and low *T*_m_, regardless of concentration of hgDNA. Single digital partition counts were observed at low-*T*_m_ non-specific amplification in both the presence of template and the NTC and independent of hgDNA concentration (Figure 9B and C). The fraction of partitions generating a false-positive amplification at low *T*_m_ was less than 3.3 × 10^−4^ through 45 min (i.e. 7 or fewer events in 20,000 partitions per chip). Similarly, partition counts of high-*T*_m_ non-specific amplification are <10 per chip until 45 min. After 90 min, high-*T*_m_ non-specific amplification is more prevalent than low-*T*_m_ non-specific amplification and the reactions finish with fewer than non-specific 260 counts in 20,000 partitions corresponding to a false-positive fraction of 1.3 × 10^−2^. One exception is the non-specific high-*T*_m_ amplification in the absence template and HHGE. This condition appears to have lower non-specific background than other conditions. We collected each replicate on separate days and are able to observe the experimental variability between the presence and absence fo template, which might be otherwise lost when examining the NTC alone. This experiment emphasizes the advantage of determining non-specific amplificaiton using *T*_m_ from the same experiment as specific amplification is counted. At low background rates, such as when using *Bst* 2.0, inherant variability exists in the false-positive fraction and can impact LOD. Measuring non-specific amplificaiton from within an experimental eliminates the assumption that the false-positive rate remains identical to the NTC or between experimental runs.

For *Bst* 3.0, non-specific amplification was variable, but tended to be fewer for higher concentrations of hgDNA. At any given time, high-*T*_m_ non-specific amplification was on average ∼30-fold more likely to occur than a low-*T*_m_ non-specific product. At 45 min, low-*T*_m_ non-specific amplification had false-positive fraction <3.1 × 10^−3^ (62 or fewer events per chip), amplification events with high *T*_m_ had a false-positive fraction <1.9 × 10^−2^ (386 or fewer events per chip). At the completion of the experiment, high-*T*_m_ non-specific amplification events account for as much as 35% of the total partitions per chip; a value exceeding the total observed true-positive events. In these scenarios, utilization of *T*_m_ to identify true and false amplification will be critical to successful quantification of target analytes.

For this CT primer set, both *Bst* 2.0 and *Bst* 3.0 similarly demonstrate that the presence of high concentrations of hgDNA may suppress the likelihood of non-specific amplification occurring. In general, for this primer set and target, we find that *Bst* 2.0 performs significantly better than *Bst* 3.0 as a consequence of having higher probability of detecting a target molecule and low likelihood of generating a non-specific amplification event.

Fourth, we asked is maximum rate impacted by the concentration of hgDNA? We hypothesize that background hgDNA may compete for the binding site of the polymerase with the target DNA or generate competing amplification events and thus, decrease the maximum observed velocity in a given partition. This phenomena would be challenging to untangle in bulk. We find that maximum rates are similar for a given enzyme, until 5000 HHGE per μl for *Bst* 2.0 (Supplementary Figure S11A) and above 100 HHGE per μl for *Bst* 3.0 (Supplementary Figure S11B). Thus demonstrating that high concentrations of HHGE may slow the rate of amplification. Furthermore, in general, and echoing the conclusions of Figure 6G and I, we observe that *Bst* 2.0 has faster maximum rate than *Bst* 3.0, regardless of the hgDNA concentration.

Fifth, we asked how is LOD impacted by the concentration of hgDNA? For *Bst* 2.0 (Supplementary Figure S11E), the LOD at a given time was similar for concentrations <5000 HHGE per μl. While the LOD in the presence of 5000 HHGE per μl was slightly worse from the detection of fewer target molecules (e.g. 0.7 versus 0.5 cp/μl at 45 min). As previously, incorporation of HRM into the final assay does not impact the LOD when using *Bst* 2.0. When using *Bst* 3.0 (Supplementary Figure S11F) and HRM to remove non-specific amplification, LOD tracks with the number of true-positive events. Thus, LOD becomes worse when efficiency is lower (i.e. at 5000 HHGE per μl). Similarly, when HRM is not incorporated in the assay, higher concentrations of HHGE tend to result in a worse LOD. However, at long amplification times, high concentrations of HHGE suppress non-specific amplification more than specific amplification, resulting in LOD enhancement relative to low concentrations of HHGE.

Cumulatively, these data show high background DNA may reduce the probability of detecting a specific molecule (analytical sensitivity), suppress the false-positive fraction (analytical specificity), reduce the velocity of amplification, and delay the start of amplification at clinically relevant concentrations of hgDNA. Thus, we conclude background hgDNA impacts dLAMP for this primer set. Generally, investigators should examine their own primer sets in the presence of high concentrations of hgDNA and take caution when examining clinical samples with high leukocyte concentrations (as reported by urinanalysis). For example, CT infection is not inherently associated with high concentrations of leukocytes and many infections are asymptomatic. Ultimately, these experiments underscore the value of quantifying non-specific amplificaiton variability, using HRM, from within the same experiment as a target is quantified. Because non-specific amplificaiton is measured within a given sample, one no longer needs to assume it remains identical to the NTC or between experimental runs.

## CONCLUSION

We predict that the combination of HRM and real-time dLAMP will be invaluable for answering many questions across a wide variety of applications, and thus our approach was designed to be accessible to most standard labs. We employed commercial chips for digitization, a commercial thermoelectric unit for heating and cooling, a commercial microscope for optical analyses and we made our data-processing script freely available. Our intention was to design an accessible system with readily available components to enable others to access the advantages of digital microfluidics to study and optimize primer sets, enzymes, and reaction conditions of interest to them. We predict these capabilities will be particularly valuable for people working with variable sample matrixes, high background DNA, poorly performing primer sets, or poorly performing enzymes.

We derived four major lessons from this study. First, LAMP can produce non-specific amplicons with high *T*_m_. The formation of these non-specific amplicons occurs from the interaction of multiple primers and the use of a polymerase with template switching ability, terminal transferase activity and lacking 3′–5′ exonuclease activity. Interaction of primers may lead not only lead to rising background fluorescence (37), but to spontaneous exponential amplification as well. Primer design and enzyme selection therefore should be judicious to avoid formation of hairpins within primers, as well as microhomology at the 3′ with any other primer, in order to prevent non-specific amplification.

Second, HRM in LAMP is a useful method for differentiating specific and non-specific amplification events. Digital experiments measure the fate and rate of each template, in contrast, bulk experiments are biased toward early amplification events. The combination of dLAMP and HRM allows observation of many amplification events and assignment of the nature of that amplification as true or false. Further, dLAMP with HRM quantifies non-specific amplification experimentally in the presence of specific amplification, eliminating the assumption that incidence of false positives in the presence of template remains identical to the NTC or between experimental runs.

Third, by differentiating specific and non-specific amplification, HRM is helpful in determining the combination processing and assay parameters that will lead to the best LOD in a digital assay. When HRM is incorporated into a dLAMP assay, true and false-positive amplification events can easily be separated. LOD is improved by elimination of non-specific background and thus becomes dependent on the number of molecules that amplify (i.e. amplification efficiency or fraction of copies detected), without dependence on the incidence of false positives. In contrast, if HRM were employed in a bulk reaction, the LOD would still be limited by the competition between specific and non-specific amplification (which amplifies first) and would require a high number of trials to achieve sufficient statistical power. Importantly, even when HRM will not be used in the final assay, it can still be incorporated during the assay-development stage to improve the assay's LOD by determining the optimal choice of parameters based on rate, TTP, final intensity or any combination of these parameters. Furthermore, our mathematical description of LOD is generalizable to other amplification methods that are measured in digital and can separate specific and non-specific amplification.

Fourth, high levels of non-specific host gDNA suppress analytical sensitivity and specificity, reduce amplification velocity, and delay the start of amplification. However, low-to-moderate levels of non-specific host gDNA do not impact the analytical specificity or sensitivity of dLAMP. We ran our assays through clinically relevant concentrations of background DNA and did not observe interference until the upper range of concentrations expected to cause interference to demonstrate the clinical utility of real-time dLAMP with HRM.

Real-time dLAMP with HRM will enable the mechanistic optimization of primers and myriad assay conditions (such as buffer, Mg^2+^ and reaction temperature). Because real-time dLAMP with HRM reveals the incidence of non-specific amplification products with high and low *T*_m_ as a function of time, dLAMP with HRM can be used to investigate approaches that will eliminate different non-specific products. For example, fast or early non-specific events in digital may indicate primers or conditions that will be especially vulnerable to failure in a bulk reaction. Thus, real-time dLAMP with HRM could be used to design primers that will suppress non-specific amplification in bulk, by generating only non-specific amplicons that occur at slow rates and late TTP.

Future efforts should investigate the combination of real-time dLAMP (and other digital isothermal amplification technologies) and HRM as a way to increase multiplexing of dLAMP when using a single reporter. In PCR, HRM has been used to differentiate among multiple amplification products by measuring differences in *T*_m_ (42–46), with applications that include among others multiplexed pathogen identification and antibiotic susceptibility testing. Finally, studies with clinical samples should be performed using the dLAMP with HRM method to understand the carryover effects from relevant matrices.

## DATA AVAILABILITY

The complete sequencing data generated during this study are available in the National Center for Biotechnology Information Sequence Read Archive repository with the BioProject ID: PRJNA574638.

The MATLAB script described here has been deposited in the open-access online repository GitHub and may be accessed using the following direct link: https://github.com/IsmagilovLab/Digital_NAAT_2Ch_MeltCurve_Analyzer.

## Supplementary Material

gkaa099_Supplemental_Files

## Notes

**Disclaimer:** The U.S. Government is authorized to reproduce and distribute reprints for Governmental purposes notwithstanding any copyright notation thereon. The views and conclusions contained herein are those of the authors and should not be interpreted as necessarily representing the official policies or endorsements, either expressed or implied, of the U.S. Government.
