# Supplementary material for: Real-time kinetics and high-resolution melt curves in single-molecule digital LAMP to differentiate and study specific and non-specific amplification
Source: Nucleic Acids Res. 2020 Feb 27;48(7):e42. doi: 10.1093/nar/gkaa099 (PMC7144905; doi:10.1093/nar/gkaa099)
Supplement: gkaa099_Supplemental_Files [file gkaa099_supplemental_files.zip › Supplement_dLAMP_rev_1.8.20.pdf]

Supplemental information for

Real-time kinetics and high-resolution melt curves in single-molecule digital LAMP to differentiate and study specific and nonspecific amplification

Justin C. Rolando,[a] Erik Jue,[b] Jacob T. Barlow,[b] and Rustem F. Ismagilov[ab]*

[a]Division of Chemistry and Chemical Engineering
[b]Division of Biology and Biological Engineering
California Institute of Technology 1200 E. California Blvd., Pasadena, CA, 91125 United States
* Correspondence to: rustem.admin@caltech.edu

Contents:

Supplementary Materials and Methods
- Primer Sequences
- Fabrication of thermoelectric unit mount
- MATLAB script
- Propagation of LOD uncertainty
  - Equations S1-S2

Supplementary Results
- *Impact of buffer conditions on specific and nonspecific amplification and Tm*
  - Figure S1
  - Table S1
- Table S2
- Figure S2
- Figure S3
- *Bulk and dLAMP reactions with modified primer sets*
  - Table S3-S4
  - Figures S4-S6
- Tables S5-7
- *Does removing outliers impact the distribution of maximum rates?*
  - Figure S7
- Figure S8
- Figure S9
- *Summary Data of Mode TTP*
  - Table S8
  - Figure S10
- Figure S11
- Tables S9-10

Caption for 4D videos

Contributions of non-corresponding authors

Supplementary Materials and Methods

Primer Sequences

Primers had the following sequences:

- BIP: AAG CAC GCG GAC GAT TGG AAA AAA GCG GAT TTG CCT AAC CG
- BOP: CGA ACA TTC CCC TTG ATC GC
- FIP: GCT GCT CCA TCG TCT ACG CAG TTT TGC TCG TCT TCC CTG GGT T
- FIP_{Short}: GCT GCT CCA TCG TCT ACG CAG TTT TGC TCG TCT TCC CTG GG
- FOP: CCA AGG TTT CCA GGG TCA A
- LoopB: CCG TAG AGC GAT GAG AAC G
- LoopF: GCC TCA ACT TAG GGG CCG

Fabrication of thermoelectric unit mount

Starting from 1/4" thick aluminum stock; a block was squared and milled to 58x61 mm and slightly less than ¼" thick. Both the side in contact with the microfluidic chips and with the thermoelectric unit were finished with a single pass of a ½" fly bit to generate a mirror finish. Four holes for screws holes were counter bored to ensure the heads remained below the surface of the block and mounted to a 1.1 °C/W Half Brick DC Converter Heat Sink (AAVID, via Newark Electronics, 241214B92200G) using four #6-32, 5/8" long screws. A 7/16" hole was clearance drilled into the side of the aluminum to ¾" depth and a thermistor (TE Tech, MP-3002) was inserted and mounted using Thermal Compound (Arctic Alumina Silver Ceramic Polysynthetic). The thermoelectic unit was mounted between the aluminum block and heat sink using Thermal Compound and the screws finger tightened. Desired torque was calculated to be 0.89-7.175 ft*lbs per screw (total pressure 70-170 psi).

Once mounted, a QuantStudio chip was place on top of the block (to mimic total load on the instrument) and the PID tuned following instructions from the TC-720 Controller manual. With I&D set at zero; P was found to oscillate at 1.35 at 70 °C (the expected dLAMP temperature). The oscillation period was 6 seconds. Thus, the Proportional BW was set at 1.7*1.35 = **2.3**. The Integral gain was calculated as I = 1.2/T in min = 1.2/0.1 = **12**. The derivative gain was calculated as 0.075xT=0.075*0.1= 0.0075 min. With these settings, the observed temperature overshoot from room temperature to 70 °C at maximum output was 0.05 °C, whereas at 95 °C the observed overshoot was 1 °C.

The ability of the embedded thermocouple to accurately assess temperature of the aluminum block was verified with an independent K-type mini-thermcouple read through a General IRT659K [IR] Thermometer.

MATLAB script

The MATLAB script works as follows: First, the TIF stack containing 2-channel images of the LAMP amplification and melt curve along with a .txt file containing temperature over time data are loaded into memory. We used the first image of the ROX channel to define all of the partitions. A custom iterative thresholding algorithm was applied to detect partitions despite lighting non-uniformities, imaging artifacts, or possible debris. The size of a well was pre-defined using the areaBound parameter. For our study, we defined partitions as having areas between 20 to 45 pixels. The algorithm scans through increasing threshold sensitivities, applies the partition size filter, and combines the results into a final mask. This is repeated for each image in the stack.

In order to track the partition intensities over time, it is important to track the same partition. This is challenging because partition move due to thermal expansion during the LAMP heating and melt curve, partitions touching the edge of the image may appear or disappear from the field of view, and bubbles during the melt curve can distort image. To account for this, we applied the built-in MATLAB labeling function to the first image of the stack to assign a unique number to each partition. We assume that a partition will not translate a distance greater than its radius from frame to frame. Using this, we find the centroid of each labeled partition in the first frame and overlay this with the second frame. If a labeled centroid overlaps a partition, the entire partition is assigned the label. If not, the partition was not found and was discarded from the analysis. This method is repeated for the centroids of the second frame onto partition of the third frame and so on. On average, more than 18,000 of the 20,000 partitions were attained for analysis, which is plenty for statistical confidence.

To analyze partitions, the intensity of each partition is averaged for each frame and plotting against time for the LAMP curve. The data is smoothed using a Gaussian blur, using the gaussWinSize parameter, with window size of 5 frames. The background baseline is subtracted from the LAMP curve. It is calculated by averaging the intensities from the six frames after the temperature of the experiment reached the optimal LAMP temperature. Time to positive (TTP) was calculated as the frame at which the intensity crossed a threshold of 250 RFU, defined using the “threshold” parameter. The derivative of the LAMP curve was calculated and the maximum slope was determined for each curve.

Partitions of interest for melt curve analysis were identified by exceeding a minimum intensity or slope (rate) threshold. Once selected, the average partition intensities during HRM were determined and smoothed similarly as for the LAMP curves. Using the temperature and time data from the .txt file, the melt intensities were replotted with temperature as the X-axis. The negative derivative of the melt curve was used to calculate the peak melt temperature for each partition. We have reported other processing parameters previously (22).

The following processing parameters were used:

- mask_thresh = .08:.002:.16;
- areaBound = [20 45] Pixels;
- threshold = 250 RFU;
- gaussWinSize = 5 Frames;
- maxSlope = 200 RFU/Frame;
- maxSlopeThreshold = 30 RFU/Frame;
- time between Frames (“time_spacing”) = 30 sec;
- LAMP Start (“LAMP_start”) = Frame 1;
- LAMP End (“LAMP_end”) = Frame 185;
- melt Curve Start (“MC_start”) = Frame 194;
- melt Curve End (“MC_end”) = Frame 241.

Propagation of LOD uncertainty

The digital loading of molecules onto a chip is a Poisson process. However, because the number of counting events is large, we can assume the counting events are approximately normally distributed, parameterized by a mean and standard deviation, σ. When measured quantities are normally distributed, then the error in any derived quantities can be found with the following expression (46):

$$\sigma_x^2 = \left(\frac{\delta x}{\delta a}\right)^2 \sigma_a^2 + \left(\frac{\delta x}{\delta b}\right)^2 \sigma_b^2 + \dots \left(\frac{\delta x}{\delta n}\right)^2 \sigma_n^2$$

(S1)

In our specific scenario, the variance of the derived quantity LOD (Equation 1) can be expressed as:

$$\sigma_{LOD}^2 = \left(\frac{1}{[N_{True} - (N_{False} + 3\sqrt{N_{False}})]/N_{CI}}\right)^2 \left(\sigma_{C_{True}}\right)^2 + \left(-\frac{C_{True}}{\left([N_{True} - (N_{False} + 3\sqrt{N_{False}})]/N_{CI}\right)^2}\right)^2 \left(\sigma_{N_{True}}\right)^2 + \left((-\frac{C_{True}}{\left([N_{True} - \left(N_{False} + 3\sqrt{N_{False}}\right)]/N_{CI}\right)^2})(-\frac{1}{N_{CI}} - \frac{3}{2N_{CI}\sqrt{N_{False}}})\right)^2 \left(\sigma_{N_{False}}\right)^2$$

(S2)

Supplementary Results

Impact of buffer conditions on specific and nonspecific amplification and Tm

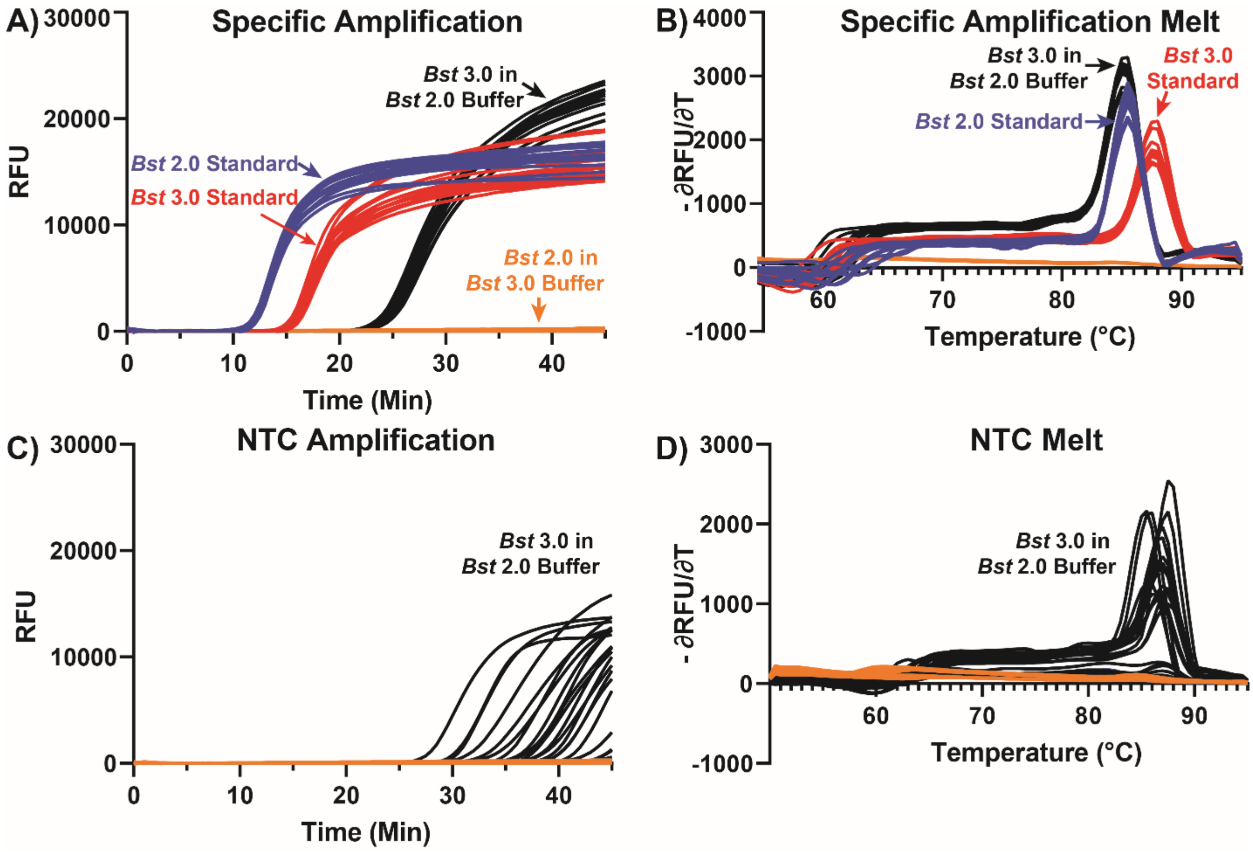

Figure S1. Amplification and melting temperature (Tm) curves of *Chlamydia trachomatis* in bulk reactions indicate enzyme sensitivity to varying buffer conditions. (A-B) Amplification curves in the presence of template (A) and Tm curves (B). (C-D) Nonspecific amplification curves in the no-template control (NTC) (C) and the associated Tm curves (D).

Table S1. Summary table of LAMP time to positive (TTP) and product melting temperature (Tm) of *Chlamydia trachomatis* amplicons under a range of buffer conditions.

Condition	Sample	TTP (Min)	Tm (°C)	Amplifications
+ Control	*Bst* 2.0, Standard (7mM Mg^{2+})	12.71±0.23	85.50±0.00	12/12
+ Control	*Bst* 3.0, Standard (8mM Mg^{2+})	16.33±0.30	87.58±0.19	12/12
+ Control, NTC	*Bst* 3.0, Standard (8mM Mg^{2+}), NTC	37.84±1.59	91.00±0.20	44/45
Switch Buffers	*Bst* 2.0, *Bst* 3.0 Buffer	N/A	N/A	0/24
Switch Buffers	*Bst* 3.0, *Bst* 2.0 Buffer	24.95±0.41	85.08±0.19	12/12
Switch Buffers, NTC	*Bst* 2.0, *Bst* 3.0 Buffer; NTC	N/A	N/A	0/24
Switch Buffers, NTC	*Bst* 3.0, *Bst* 2.0 Buffer; NTC	30.75±7.62	86.85±0.72	20/24

We next wished to determine if the behavior associated with nonspecific amplification was inherent to the polymerase or the buffer for both *Bst* polymerases. Buffer composition may influence nonspecific amplification more than the selection of polymerase. We conducted bulk reactions in the presence and absence of template using the standard buffer compositions (Materials and Methods) and the same reactions with each polymerase in the opposite buffer. When we used *Bst* 2.0 polymerase with the *Bst* 3.0 buffer, amplification failed to occur in both the presence and absence of template. When we used *Bst* 3.0 polymerase with *Bst* 2.0 buffer, we observed (i) an 8.6 min delay in TTP (from 16.33±0.30 min to 24.95±0.41 min) in the presence of template (ii) earlier nonspecific amplification in the absence of template, and (iii) greater variation in TTP (from 37.84±1.59 min to 30.75±7.62 min). From these data, we concluded that the difference in nonspecific amplification between conditions was an issue inherent to polymerase selection.

We next tested if the differences in Tm of the target amplicons were due to buffer components. We observed similar sequencing results for these products, but differing Tm. We conducted bulk reactions in the presence of template using the standard buffer composition (Materials and Methods) and the same reactions with each polymerase in the opposite buffer. When all buffer components were switched between the polymerases, *Bst* 2.0 failed to amplify, whereas *Bst* 3.0 resulted in amplicons with Tm similar to *Bst* 2.0 in standard conditions (85.08±0.19 °C). We concluded the *Bst* polymerase produced similar specific products and differences in Tm were due to differences in buffer conditions.

Table S2. List of abbreviations used in Figures 2 and 4.

Abbreviation	Long Form
BIP	Backward Inner Primer
rcBIP	Reverse Compliment of BIP
prcBIP	Partial Reverse Compliment of BIP
FIP	Forward Inner Primer
rcFIP	Reverse Compliment of FIP
pFIP	Partial FIP
loopB2	Backward Loop Primer
rcloopb2	Reverse Compliment of Backward Loop Primer
TargetDNA	CT Target DNA sequence
rcTargetDNA	Reverse Compliment CT Target DNA sequence
Rand	Random Insert

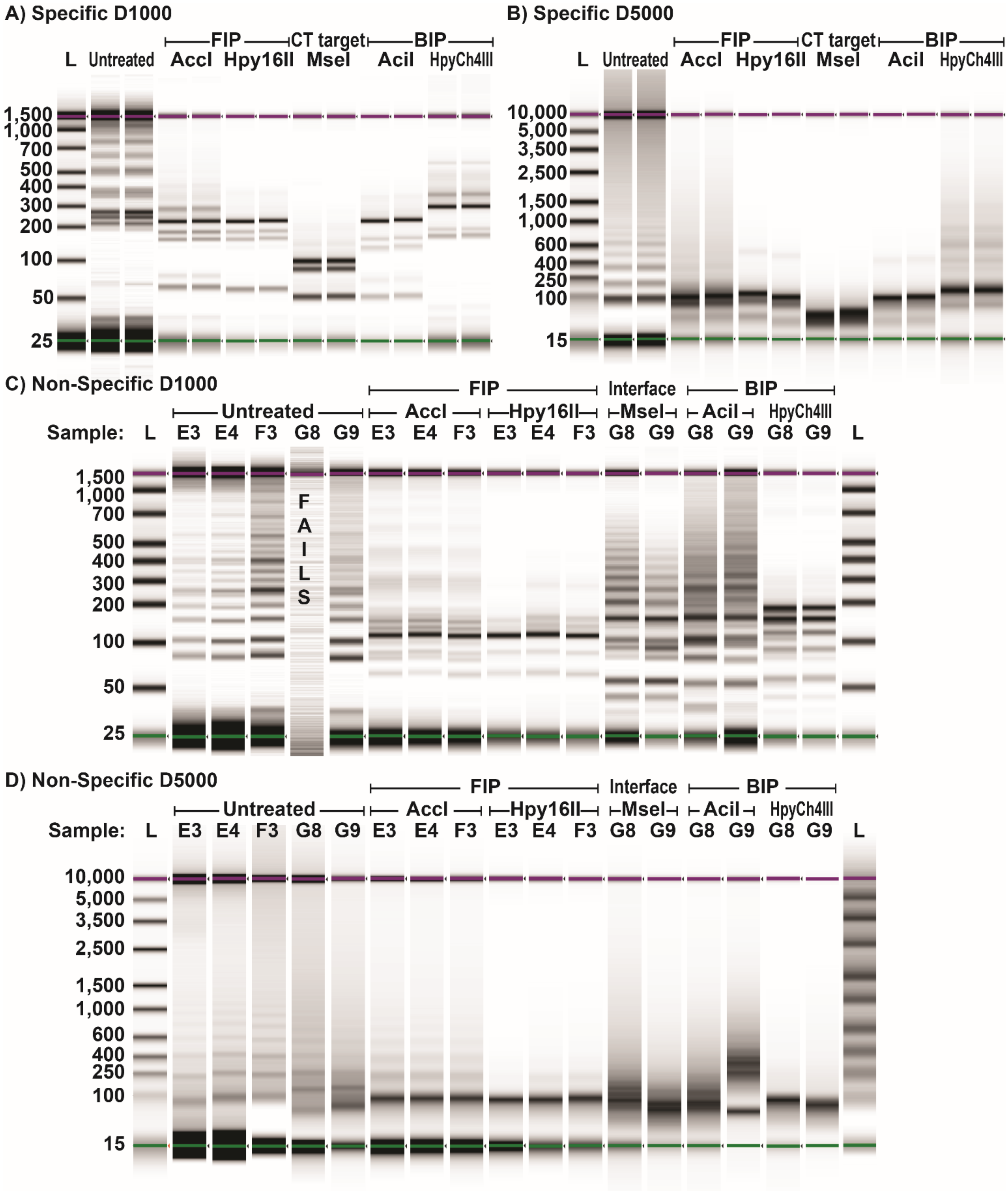

Figure S2. Composite images of restriction digestion of *Chlamydia trachomatis* (CT) bulk amplification products. Digestion of specific amplification products using a D1000 DNA ScreenTape (A) and a D5000 DNA ScreenTape (B). Digestion of nonspecific amplification products using a D1000 DNA ScreenTape (C) and a D5000 DNA ScreenTape (D). AccI and Hpy16II target restriction site in FIP, MseI in the specific products targets a region within the CT sequences, and in the presence of nonspecific amplification products targets the interface (synthesis across a discontinuous junction) of FIP and BIP. AciI and HpyCh4III target restriction endonuclease sites within BIP.

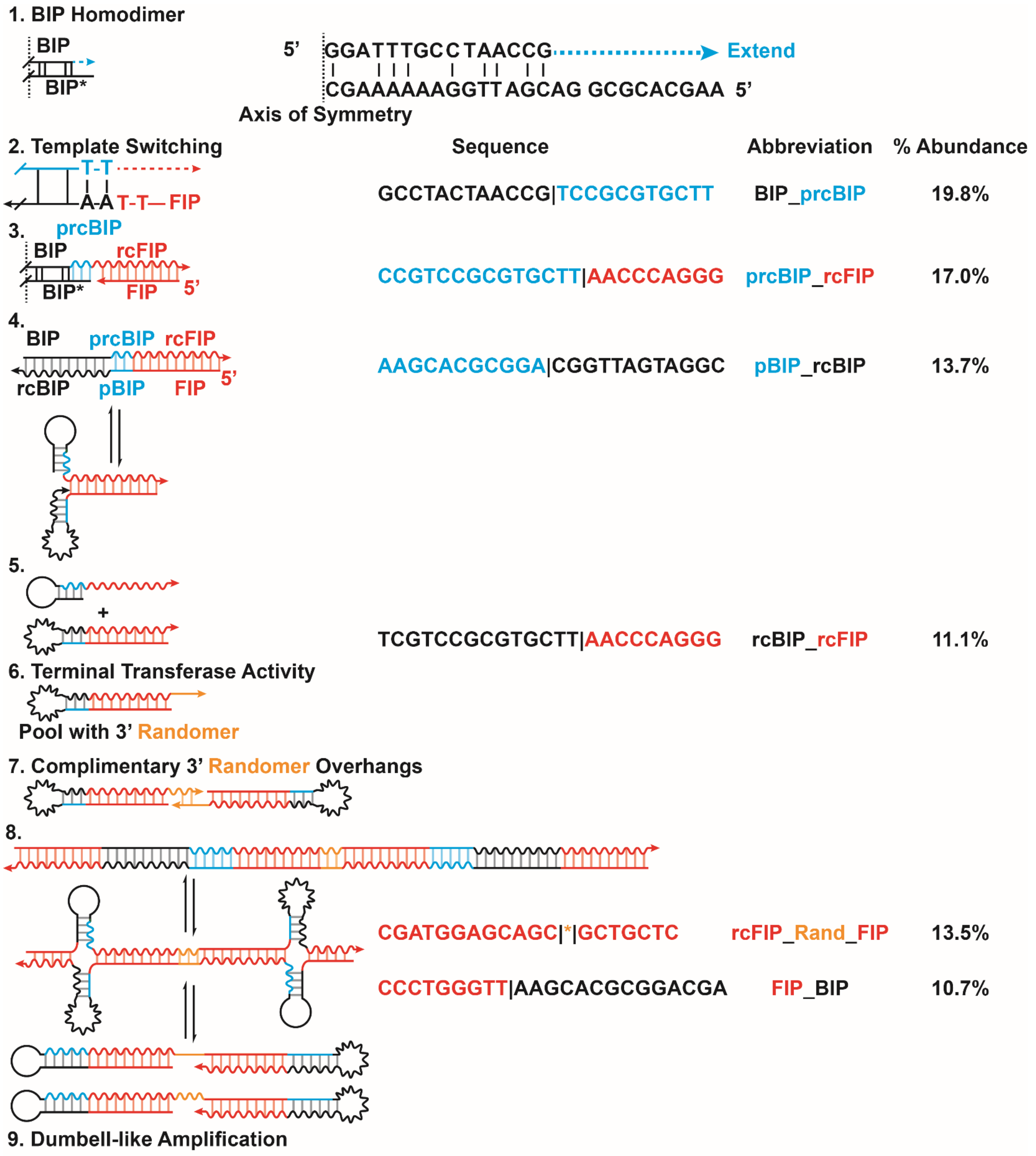

Figure S3. Illustration of a simplified mechanism for nonspecific amplification products in LAMP reactions. Structures and intermediates are labeled with numbers. Percentage abundance reported from Fig. 2, Sample E2.

Bulk and dLAMP reactions with modified primer sets

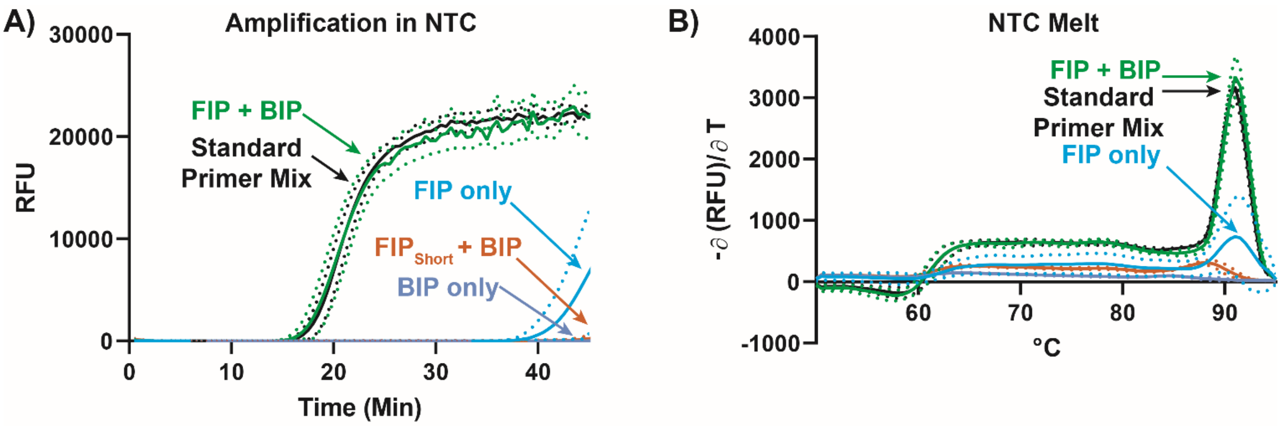

Figure S4. Amplification and melting temperature (Tm) curves of *Chlamydia trachomatis* (CT) in a bulk reaction using multiple primer sets show reduced nonspecific amplification upon elimination of primer microhomology. Plots of average fluorescence as a function of time during the LAMP reaction in the NTC (A) and the corresponding derivative plot of fluorescence as a function of temperature (B). N per condition = 12.

Table S3. Time to mean positive and Tm in bulk reactions using multiple primer sets. N per condition = 12.

Sample	Time to Positive (Min)	Tm (°C)
FIP only	42.3±1.8	91.7±0.8
BIP only	n/a	n/a
FIP + BIP only (No Other Primers)	18.1±1.1	91.0±0.1
Standard Primer Mix	18.3±0.7	91.0±0.0
FIP_{Short} + BIP	n/a	n/a

We analyzed multiple primer sets (**Table S3**) to determine if the nonspecific amplification species produced in the NTC were indeed produced from a combination of primers, as described by the sequencing data in **Fig. 2** and the mechanism proposed in **Fig. 4.** We also wished to test whether nonspecific amplification could occur by single primers, as is known to occur. We conducted bulk reactions in the absence of template using *Bst* 3.0 and the standard primer mixture, FIP and BIP in combination, and the Inner Primers alone and compared the TTP and Tm of these mixtures. The standard primer mixture (consisting of Inner, Outer, and Loop primers) had nonspecific amplification at 18.3±0.7 min with uniform Tm of 91.0±0.0 °C. When the Outer and Loop primers were removed, leaving only FIP and BIP, the mixture amplified with similar TTP and Tm (18.1±1.1 min and 91.0±0.1 °C, respectively) as the standard mixture. In contrast, using BIP alone failed to amplify within 45 min, and FIP alone amplified much later (42.3±1.8 min) and with different Tm (91.7±0.8) than FIP and BIP together or the standard primer mixture. We thus concluded that both FIP and BIP were required to generate the nonspecific products we observed.

The mechanism proposed in **Fig. 4** requires an interaction between BIP and FIP via microhomology of the 3' of FIP. To confirm the suspected interaction between FIP and BIP, we removed two bases from the 3' end of the FIP primer (hereafter FIP_{Short}). In bulk reactions using FIP_{Short} and BIP with *Bst* 3.0 in the absence of template, we did not observe nonspecific amplification within 45 min. Consequently, we concluded, some of the nonspecific amplification was due to an interaction of the 3' of FIP with BIP.

We next ran the modified primer set in digital LAMP using *Bst* 3.0 to improve our understanding of what occurs at the single-molecule level when primer microhomology is eliminated. We ran three chips in the presence of template and three chips in the absence of template, using the standard primer set (**Fig. S4A**), and compared the results to the same experiments run with a primer set with FIP_{Short} (**Fig. S4B**). We observed a significant increase in the percentage of copies detected when using FIP_{Short} (**Fig. S4C**) using a two-tailed paired t-test (P = 0.002), without a difference in TTP (**Table S3**).

The use of FIP_{Short} did not significantly impact nonspecific amplification products with low Tm in any pairwise ANOVA comparison (**Fig. S4D,E**). However, we observed a 10-100 fold decrease in nonspecific products with high Tm (**SI Fig. S4F, G**). In the absence of template, nonspecific amplification was reduced (**Fig. S4E**); whereas in the presence of template, the number of nonspecific amplification products with high Tm was significantly lower at all time points (**Fig. S4G**).

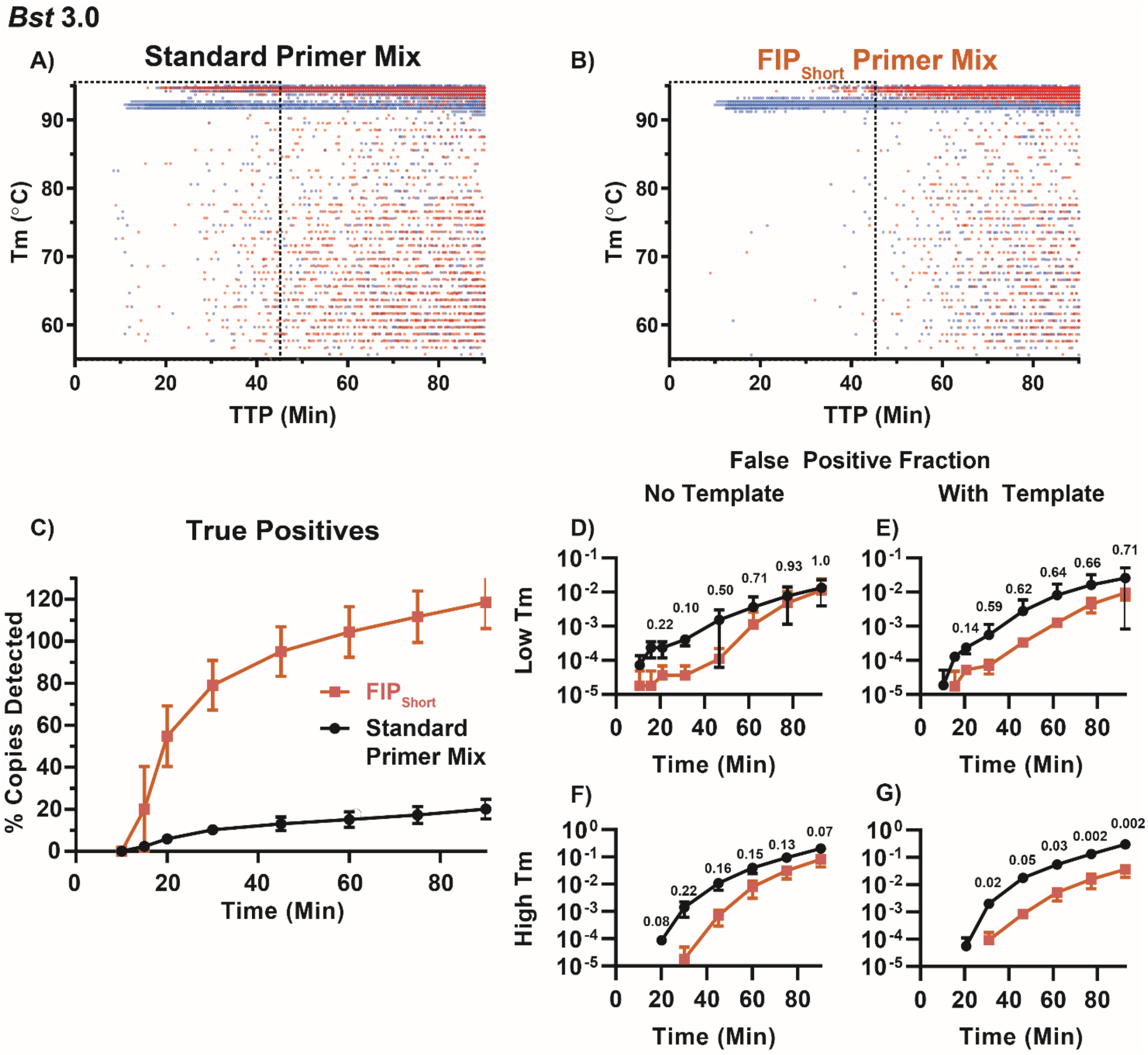

Figure S5. Digital, single-molecule plots of specific and nonspecific amplification for multiple primer sets of *Chlamydia trachomatis* show significantly reduced nonspecific amplification with high melting temperature (Tm) upon elimination of primer microhomology. (A-B) Individual amplification events using *Bst* 3.0 and a primer mixture where FIP is replaced with FIP_{Short} (A) and the standard primer mixture (B). Blue indicates amplification events in the presence of template; red indicates amplification in the absence of template (NTC). A box is placed around events that occur within 45 min, corresponding to the bulk amplification time. Partitions are rendered at 50% opacity. (C) Plot of the percentage of copies detected (specific amplification) as a function of time. (D-E) The fraction of partitions with nonspecific amplification with Tm less than the specific amplification as a function of time in the absence of template (D) and in the presence of template (E). (F-G) The fraction of partitions with nonspecific amplification with Tm greater than the specific amplification in the NTC as a function of time (F) and in the presence of template (G). Within panels D-G, pairwise *P*-values by t-test are written above each time point.

Table S4. Digital, single-molecule comparison of specific and nonspecific amplification for multiple primer sets of *Chlamydia trachomatis* using *Bst* 3.0.

	Time to mode positive (min)
Standard Primer (Set 1)	15.7±2.7
(Set 2)	13.7±0.9
Modified Primer	15.0±1.7

The decrease in nonspecific products with high Tm, upon elimination of the microhomology between FIP and BIP, is consistent with the formation of a nonspecific product predicted by the proposed mechanism in **Fig. 4**. However, the continued existence of nonspecific products indicates it is possible to form a variety of nonspecific products. Our results indicate that nonspecific products with high-Tm occur even with further primer optimization. The formation of products with high Tm is consistent with our proposed mechanism of branched, mesh like network. Further investigation should determine if this problem is ubiquitous, even in optimized systems. Additionally, the delay in nonspecific amplification in digital, could explain why we did not observe these products in bulk (and thus cannot sequence them).

We believe the mechanism described in **Fig. 4** is potentially applicable to other primer sets. Amplification observed by FIP alone may follow a similar amplification scheme to **Fig. 4** via homo-dimerization (**Fig. S6A**), non-templated synthesis, hairpin dimerization (**Fig. S6B**), and eventually dumbbell-like amplification (**Fig. S6D,E**). We observe products consistent with these structures in some of the sequencing data (e.g. **Fig. 2**, Well E1, which contains elevated rcFIP_pFIP and rcpFIP_FIP).

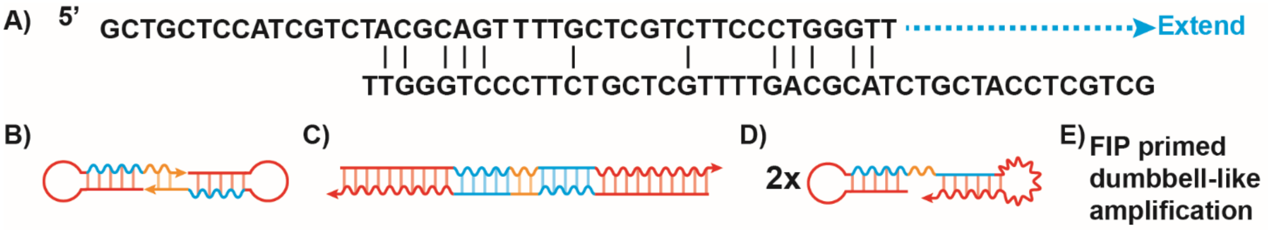

Figure S6. Illustration of a mechanism for amplification of FIP alone. After 3' extension of a FIP homodimer (A), random nucleotides may be incorporated (orange) resulting in self-complimentary hairpins (B). The extension of these hairpins products product (C): top strand, FIP-prcFIP-rand-pFIP-rcFIP. Upon melting, two self-amplifying dumbbell structures can be produced (D), and undergo further LAMP-like amplification primed by FIP (E).

Table Group S5- NGS of Randomer inserts within Bulk Sample E1.

E1	
Length	Percent
4	53.1
2	41.0
3	2.6
5	1.8
6	1.1

E1: Length n=4	
Sequence	Percent
CGTT	38.6
AACG	33.1
AGTT	8.1
AACT	7.5
GGTT	2.5
AACC	2.5
CGTC	1.4
GACG	1.2

E1: Length n=2	
Sequence	Percent
AT	28.4
GT	15
AC	13.8
CC	10.3
GG	10.2
CT	9.1
AG	8.3
GC	3.8

Table Group S6- NGS of Randomer inserts within Bulk Sample E2.

E2	
Length	Percent
3	53.4
11	13.8
4	12.7
2	9.0
5	3.7
8	2.9
14	1.7

E2: Length n=3	
Sequence	Percent
CGT	17.9
ACG	16.5
ACT	12.6
AGT	12.4
ACC	7.2
GGT	6.5
CCC	6.1
GGG	5.3

E2: Length n=11	
Sequence	Percent
AGCAGCAGCAG	20.2
CTGCTGCTGCT	18.9
ATGCTGCTGCT	19.7
AGCAGCAGCAT	18.4
AGCAGCAGCAC	4.7
GTGCTGCTGCT	4.6

Table Group S7- NGS of Randomer inserts within Bulk Sample F1.

F1	
Length	Percent
5	47.9
3	32.1
4	16.6
2	2.4

F1: Length n=5	
Sequence	Percent
GTTGT	17.8
ACAAC	16.3
GTTGC	15.3
GCAAC	13.4
ATTGC	8.4
GCAAT	8.4
ATTGT	7.7
ACAAT	7.2

F1: Length n=3	
Sequence	Percent
CGT	14.8
ACG	13.7
CGC	13.7
GCG	12.3
AGC	8.9
GCT	8.3
ACT	8.3
AGT	8.3

Does removing outliers impact the distribution of maximum rates?

Occasionally, we observed outlier data points in maximum rate. We asked what caused one point (green circle, max rate 56 RFU, **Fig. S2A**) to separate from the majority of the data (17 to 30 RFU/30 sec), if these points were common, and if these points were likely to misrepresent the max rate data. We determined the individual trace corresponding to the outlier amplification event (green trace, **Fig. S2B**) and observed that the maximum rate for this partition was at 52.5 min, corresponding to a fluctuation in the plateau phase of amplification (dotted line).

We hypothesized that the maximum rate should occur at the observed initial moment of exponential amplification, often slightly before the fluorescence TTP threshold (250 RFU) is reached. To test this hypothesis, we determined the frame (2 per minute) where the amplification trace reached the TTP. From this frame we subtracted the frame where maximum rate was calculated and plot it against maximum rate (**Fig. S2C**). Values greater than zero represent partitions where the frame the maximum rate occurs before the fame of TTP, while negative values occur when fame the max rate occurs is after the frame of TTP. We draw a vertical line separating partitions that occurring more than 15 min after the TTP (left), from all other partitions.

For the case of *Bst* 2.0, we observed that the mode max rate occurred before the fluorescence TTP by 1 frame (30 sec). Of the 9099 partitions exceeding the 250 RFU threshold, 821 (9.02%) were more than 15 min after the TTP. We expect these partitions to have max rate within the noise of the plateau phase.

A similar trend was observed for *Bst* 3.0 (**Fig. S2D**). With *Bst* 3.0, the mode max rate occurred 2 frames before the fluorescence TTP (1 min). This value is later than *Bst* 2.0 and is consistent with a slower max rate for *Bst* 3.0 than for *Bst* 2.0. Of the 24,466 partitions reaching the 250 RFU threshold, 1113 (4.55%) were more than 15 min after TTP.

To determine whether removing the partitions with max rate more than 15 min after the TTP impacted the distribution of enzymatic rates, we plotted the fractional cumulative distribution function (CDF) of max rate for all partitions (blue), and the same fractional CDF removing those points more than 15 min after the fluorescence intensity based TTP (red) for *Bst* 2.0 (**Fig. S2E**) and *Bst* 3.0 (**Fig. S2F**). Performing a non-parametric based Kolmogorov–Smirnov test to compare the exclusion of partitions with late max rate indicated non-significance between the two CDFs (*Bst* 2.0 P=0.3255, and *Bst* 3.0 $P = 0.1236$). Thus, we concluded removing late max rate data from the distributions did not impact the CDFs, and therefore does not significantly impact the integrity of our data reporting.

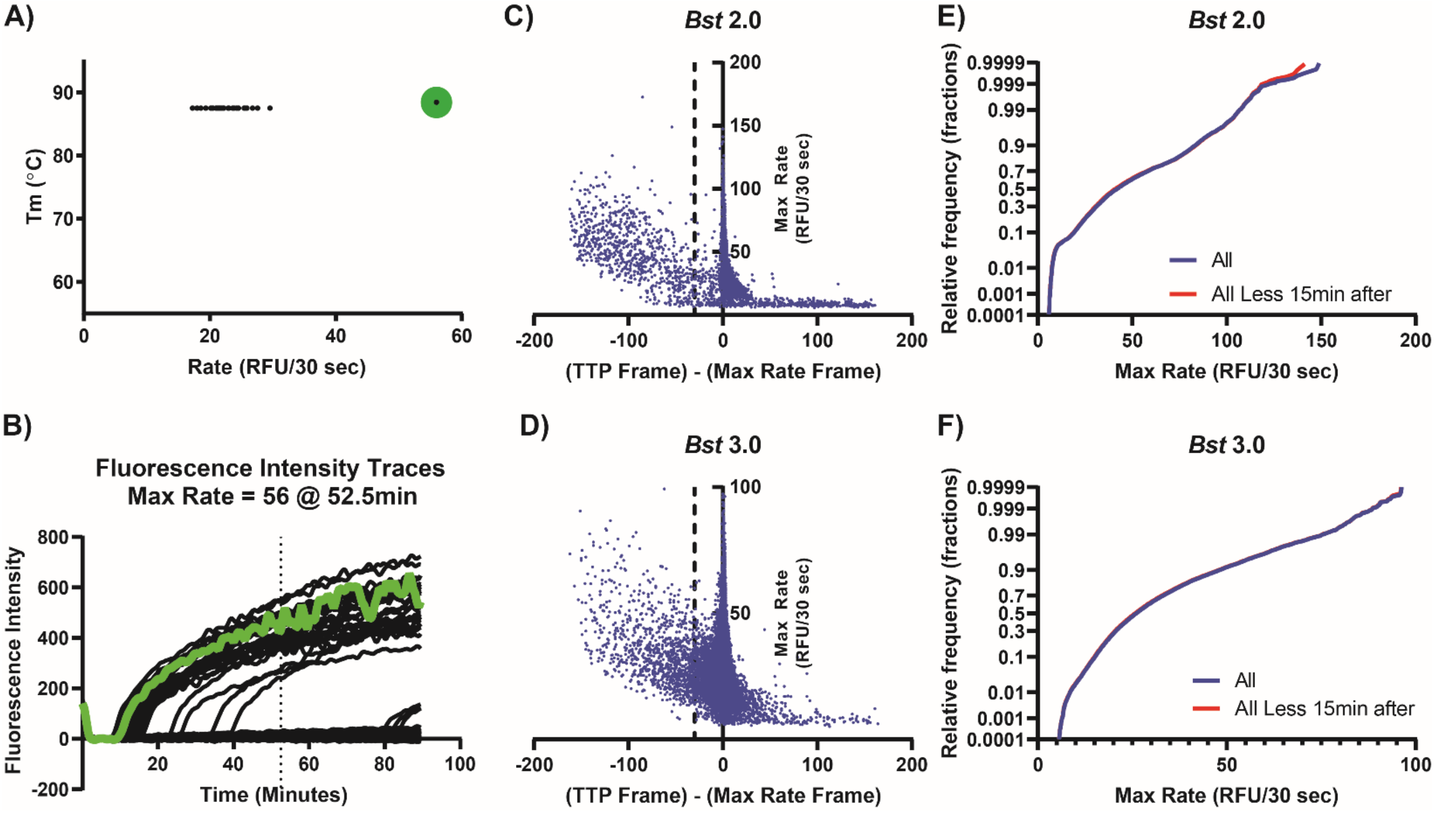

Figure S7. Removing outlier data in max rate does not significantly impact summary data. Plot of observed melting temperature (Tm) as a function of maximum rate, with a possible outlier point highlighted in green (A). Fluorescence traces of individual partition amplification events, with the possible outlier partition's trace highlighted in green (B). The maximum rate for the green trace occurred at 52.5 min (dotted line), corresponding to a fluctuation in the plateau phase of amplification. Plot of maximum rate as a function of the difference between the TTP and max rate frames using *Bst* 2.0 (C) and *Bst* 3.0 (D). Partitions lower than the dashed vertical line represent partitions whose max rate occurred more than 15 min after the TTP frame. Fractional Cumulative Distribution Plots of maximum rate for *Bst* 2.0 (E) and *Bst* 3.0 (f), where the CDF includes all possible partitions (blue), and the same fractional CDF removing those points more than 15 min after the Fluorescence Intensity based TTP (red).

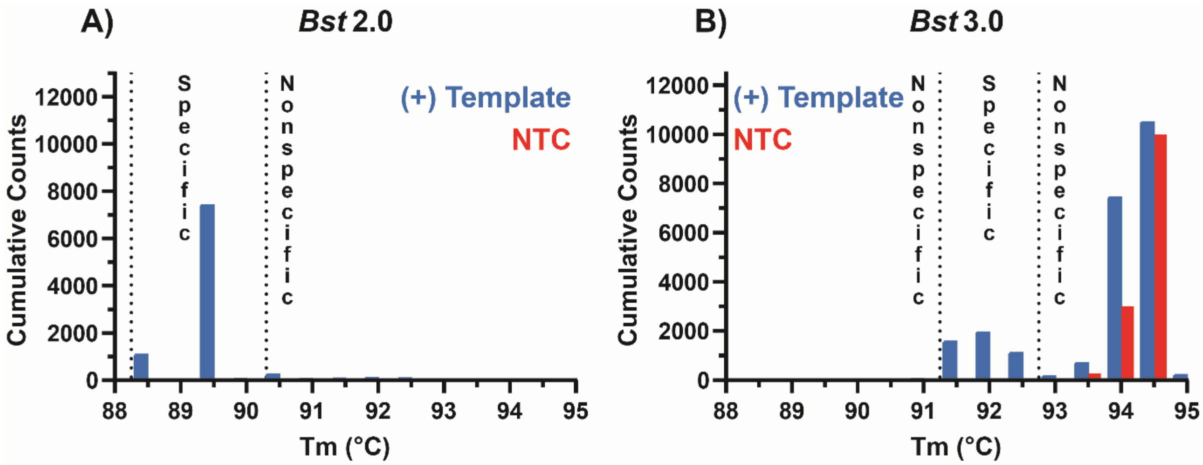

Figure S8. Histogram plots of Tm after 90 min of digital LAMP targeting CT in the presence of template (blue) and NTC (red) can be used to distinguish specific from nonspecific amplification. Tm of amplification using *Bst* 2.0 (A) and *Bst* 3.0 (B). Dashed lines indicates the upper and lower bounds used for separating specific and nonspecific amplification. *Bst* 2.0: 88.5-90.3°C, *Bst* 3.0: 91.25-92.75 °C. Bin width in both graphs 0.5 °C, with the (+) template left of the tick and NTC right of the tick. NTC is illustrated with red bars, and the presence of template with blue bars.

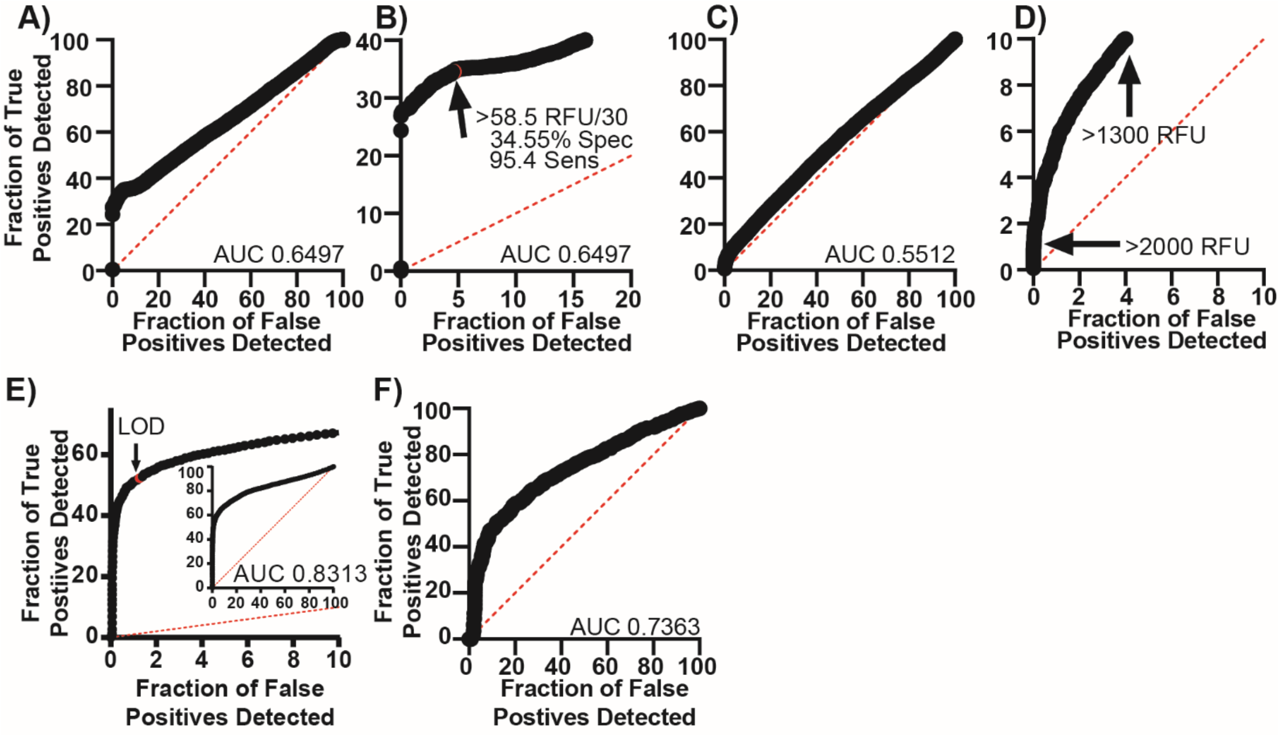

Figure S9. Receiver operating characteristic (ROC) curves using HRM to determine optimal performance of dLAMP assays. A) ROC curve using *Bst* 3.0, plotting the fraction of true positives detected versus the fraction of false positives detected using a threshold on max rate. B) ROC curve using *Bst* 3.0, plotting the fraction of true positives detected less than 40% versus the fraction of false positives detected less than 20% using a threshold on maximum rate. Arrow indicates corresponding LOD. C) ROC curve using *Bst* 3.0, plotting the fraction of true positives detected versus the fraction of false positives detected using a threshold on final intensity of the partition. D) ROC curve using *Bst* 3.0, plotting the fraction of true positives detected less than 10% versus the fraction of false positives detected less than 10% using a threshold on final intensity of the partition. Arrows indicate final-intensity thresholds of >2000 RFU and >1300 RFU. E) ROC curve using *Bst* 3.0, plotting the fraction of true positives detected versus the fraction of false positives detected using a threshold on TTP. Arrow indicates LOD. F) ROC curve using *Bst* 2.0, plotting the fractions of true versus false positives detected using a threshold on TTP.

Summary Data of Mode TTP

Table S8. Time to mode positive in minutes. N=3 chips per set. Human Haploid Genome Equivalents (HHGE) are per microliter.

HHGE per µL	0	0.01	1	100	5000
Bst 2.0	11.8 ± 0.2	11.7±0.2	11.3±0.2	11.7±0.6	16.3±2.7
Bst 3.0 (Set 1)<br>(Set 2)	15.7± 2.7<br>13.7± 0.9	13.8±1.2	18.2 ±4.4	14.7± 1.7<br>17.2± 2.1	 17.2± 2.1

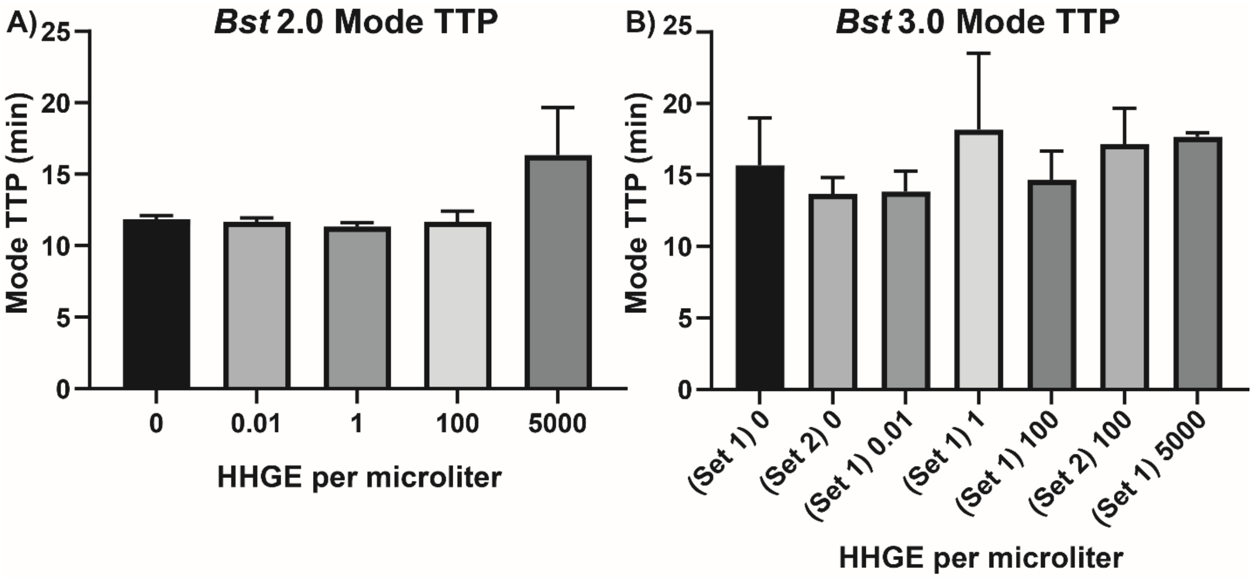

Figure S10. Time to mode positive for *Bst* 2.0 (A) and *Bst* 3.0 (B) under variable concentrations of host human genomic DNA (hgDNA). Human Haploid Genome Equivalents (HHGE) are per microliter.

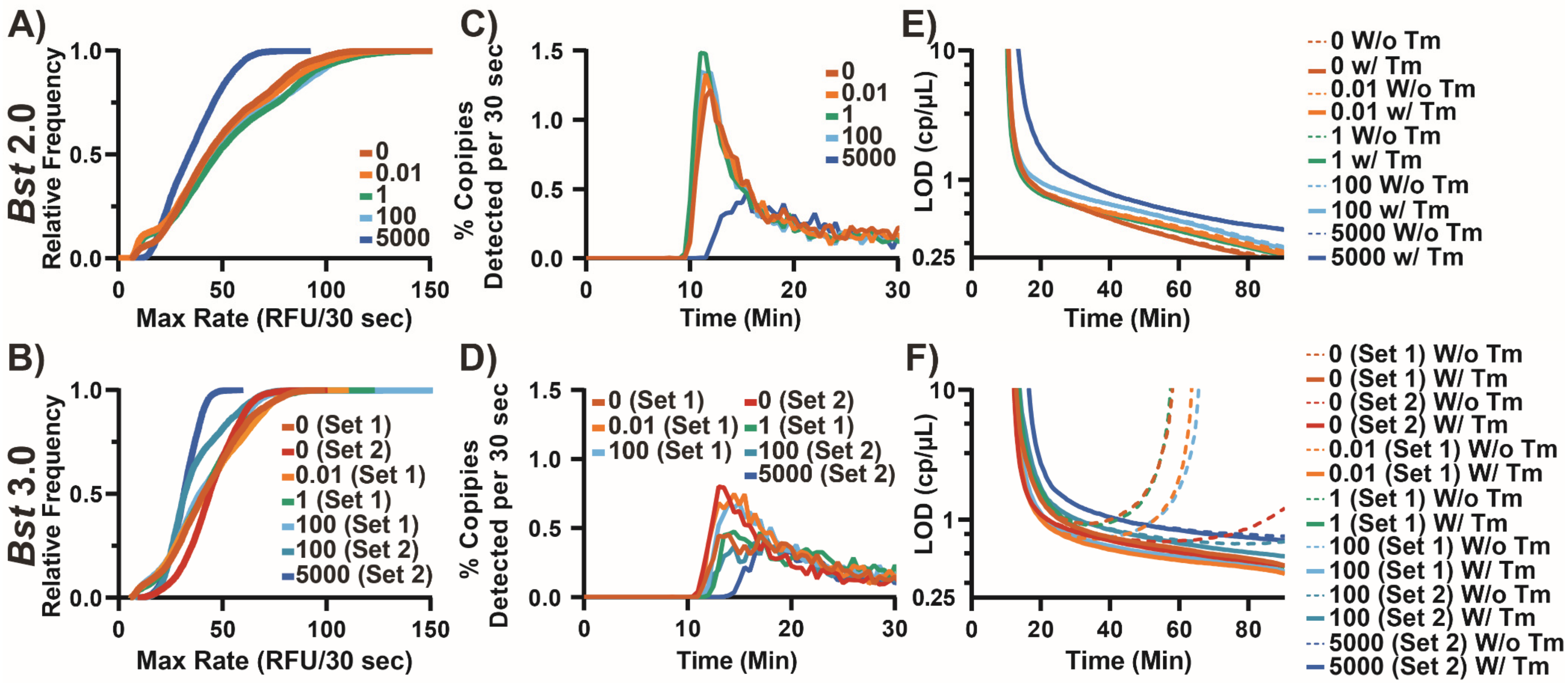

Figure S11. Evaluation of the impact of gDNA on assay performance. Fractional cumulative distribution function (CDF) of maximum rates of amplification using *Bst* 2.0. (A) and *Bst* 3.0 (B) CDFs are plotted as the sum of replicates. Distribution plot of time to fluorescence threshold for *Bst* 2.0 (C) and *Bst* 3.0 (D) using arithmetic mean. LOD as a function of time using *Bst* 2.0. (E) and *Bst* 3.0 (F).

Table S9. Tabular form of % copies detected from Fig. 9 using *Bst* 2.0.

Time (Min)	0 HHGE per µL	0.01 HHGE per µL	1 HHGE per µL	100 HHGE per µL	5000 HHGE per µL
10	0.01±0.02	0.00±0.00	0.04±0.03	0.02±0.02	0.00±0.00
15	8.21±0.72	8.35±0.56	9.42±0.56	8.98±0.81	1.67±2.32
20	11.92±0.35	12.04±0.20	12.86±0.54	12.34±1.44	5.24±3.69
30	15.94±1.13	15.41±0.36	16.15±0.66	15.65±1.98	9.19±2.66
45	22.20±4.37	19.30±0.85	20.16±1.12	19.84±1.73	13.42±1.99
60	28.88±8.07	23.68±2.49	24.94±3.05	25.00±1.11	16.96±2.03
75	36.10±11.86	29.93±5.30	31.24±5.83	31.81±2.83	20.34±2.17
90	43.77±14.98	37.47±8.62	38.47±10.05	40.69±5.32	23.12±2.35

Table S10. Tabular form of % copies detected from Fig. 10 using *Bst* 3.0.

Time (Minutes)	0 HHGE per µL (Set 1)	0 HHGE per µL (Set 2)	0.01 HHGE per µL (Set 1)	1 HHGE per µL (Set 1)	100 HHGE per µL (Set 1)	100 HHGE per µL (Set 2)	5000 HHGE per µL (Set 2)
10	0.00±0.00	0.00±0.00	0.00±0.01	0.00±0.00	0.00±0.00	0.00±0.00	0.00±0.00
15	2.28±1.99	4.13±0.98	3.83±2.47	1.71±1.54	2.86±1.04	1.37±1.89	0.05±0.08
20	5.96±2.47	8.15±0.31	8.93±2.44	5.21±4.50	7.89±0.66	4.81±2.04	3.04±0.93
30	10.19±2.48	11.23±0.23	13.33±1.84	10.26±3.32	12.41±0.24	8.82±1.06	6.86±1.05
45	13.02±3.28	14.07±0.80	16.74±1.87	13.92±2.80	15.67±0.38	11.60±1.12	9.35±1.09
60	15.09±3.68	16.28±1.71	19.13±1.83	16.37±2.69	17.89±0.29	13.81±1.70	10.92±1.38
75	17.25±4.02	18.38±3.13	21.00±2.20	18.61±3.05	19.95±0.45	15.62±2.34	12.11±1.16
90	20.11±4.71	20.44±4.50	23.97±3.58	21.13±3.99	23.00±0.87	17.37±3.16	13.10±1.81

Caption for 4D videos

Videos plot the TTP, max rate, final intensity, and Tm data of both specific and nonspecific amplification reactions using either *Bst* 2.0 (Video 1) and using *Bst* 3.0 (Video 2). Time to positive (TTP), max rate, and melting temperature (Tm) are plotted on the axes; final intensity is indicated by the color of each data point (scale provided in Fig. 6Q-R).

Contributions of non-corresponding authors

J.C.R. conceptualized the method, designed and ran all the experiments, generated and analyzed the data, wrote the paper, and constructed all figures, except Fig. 2, Table Groups S5-7.

E.J. wrote the MATLAB software script for automated analysis of digital LAMP image sequences.

J.T.B. Analyzed sequencing data in Fig. 2 and Table Groups S5-7.
